# Ten years of implementation outcomes research: a scoping review

**DOI:** 10.1186/s13012-023-01286-z

**Published:** 2023-07-25

**Authors:** Enola K. Proctor, Alicia C. Bunger, Rebecca Lengnick-Hall, Donald R. Gerke, Jared K. Martin, Rebecca J. Phillips, Julia C. Swanson

**Affiliations:** 1 The Brown School, Shanti Khinduka Distinguished Professor Emerita, Washington University in St. Louis, St. Louis, USA; 2 College of Social Work, The Ohio State University, Columbus, OH USA; 3 The Brown School, Washington University in St. Louis, St. Louis, USA; 4 Department of Social Work, College of Arts and Sciences, University of Alabama at Birmingham, Birmingham, USA; 5 College of Education & Human Ecology, The Ohio State University, Columbus, OH USA; 6 College of Liberal Arts & Sciences, Western Oregon University, Monmouth, OR USA; 7 Department of Psychiatry, Washington University School of Medicine, St. Louis, MO USA

**Keywords:** Acceptability, Adoption, Appropriateness, Feasibility, Fidelity, Implementation cost, Penetration, Sustainability, Implementation outcome

## Abstract

**Background:**

Proctor and colleagues’ 2011 paper proposed a taxonomy of eight implementation outcomes and challenged the field to address a research agenda focused on conceptualization, measurement, and theory building. Ten years later, this paper maps the field’s progress in implementation outcomes research. This scoping review describes how each implementation outcome has been studied, research designs and methods used, and the contexts and settings represented in the current literature. We also describe the role of implementation outcomes in relation to implementation strategies and other outcomes.

**Methods:**

Arksey and O’Malley’s framework for conducting scoping reviews guided our methods. Using forward citation tracing, we identified all literature citing the 2011 paper. We conducted our search in the Web of Science (WOS) database and added citation alerts sent to the first author from the publisher for a 6-month period coinciding with the WOS citation search. This produced 1346 titles and abstracts. Initial abstract screening yielded 480 manuscripts, and full-text review yielded 400 manuscripts that met inclusion criteria (empirical assessment of at least one implementation outcome).

**Results:**

Slightly more than half (52.1%) of included manuscripts examined acceptability. Fidelity (39.3%), feasibility (38.6%), adoption (26.5%), and appropriateness (21.8%) were also commonly examined. Penetration (16.0%), sustainability (15.8%), and cost (7.8%) were less frequently examined. Thirty-two manuscripts examined implementation outcomes not included in the original taxonomy. Most studies took place in healthcare (45.8%) or behavioral health (22.5%) organizations. Two-thirds used observational designs. We found little evidence of progress in testing the relationships between implementation strategies and implementation outcomes, leaving us ill-prepared to know how to achieve implementation success. Moreover, few studies tested the impact of implementation outcomes on other important outcome types, such as service systems and improved individual or population health.

**Conclusions:**

Our review presents a comprehensive snapshot of the research questions being addressed by existing implementation outcomes literature and reveals the need for rigorous, analytic research and tests of strategies for attaining implementation outcomes in the next 10 years of outcomes research.

**Supplementary Information:**

The online version contains supplementary material available at 10.1186/s13012-023-01286-z.

Contributions to the literature

- This scoping review illustrates how research on implementation outcomes in health and behavioral health settings has grown over the last 10 years, and which implementation outcomes and study contexts are understudied.
- Literature increasingly reports and describes implementation outcomes. However, few studies report hypothesis-based tests or models showing how implementation outcomes can change, be intervened upon, or affect other outcomes.
- The role of implementation outcomes in signaling inequity or advancing equity is virtually ignored.
- Findings point to the need for future theory-building research that examines interrelationships among implementation, service, and client outcomes.

## Background

Implementation outcomes reflect the progress towards success of efforts to implement evidence-based innovations. More specifically, they help disentangle the complex process of implementation so we can identify and target intermediate outcomes that may influence an intervention’s success in context. Robust conceptualization and rigorous measurement of these outcomes are essential for precision in implementation research, including understanding and testing the effectiveness of implementation strategies and explaining their mechanisms of action.

A 2011 paper by Proctor and colleagues advanced the concept of implementation outcomes, identified their critical role in implementation evaluation, and distinguished them from other traditionally measured outcomes (service system and clinical outcomes) [1]. The authors proposed a heuristic taxonomy of implementation outcomes and challenged the field to address a two-pronged research agenda: advance conceptualization and measurement and build theory including the identification and testing of change mechanisms. Ten years since the taxonomy’s publication, this paper maps the field’s progress in response to the originally proposed research agenda.

### Conceptualization of implementation outcomes in the 2011 paper

Proctor and colleagues identified eight implementation outcomes [1] acceptability is defined as stakeholders’ perceptions that an implementation target is agreeable, palatable, or satisfactory. Adoption (also called uptake) is the intent, initial decision, or action to employ an implementation target. Appropriateness is the perceived fit, relevance, or compatibility of an implementation target for a given context or its perceived fit for a problem. Feasibility is the extent to which an implementation target can be successfully used or deployed within a given setting. Fidelity is the degree to which an intervention was implemented as prescribed or intended. Implementation cost is the financial impact of an implementation effort and must become bearable for implementation to proceed. Penetration—the integration or saturation of an intervention within a service setting and its subsystem—is calculated as a ratio of those to whom the intervention is delivered divided by the number of eligible or potential recipients. Sustainability is the extent to which an implementation target is maintained or institutionalized within a service setting. The 2011 paper encouraged further scholarship of this initial conceptualization, both in terms of the number of outcomes and in further refinements to their operationalization [1]. Cautioning that the original taxonomy included “only the more obvious”, that paper projected that new concepts would emerge as newly defined implementation outcomes [1].

**Fig. 1:**
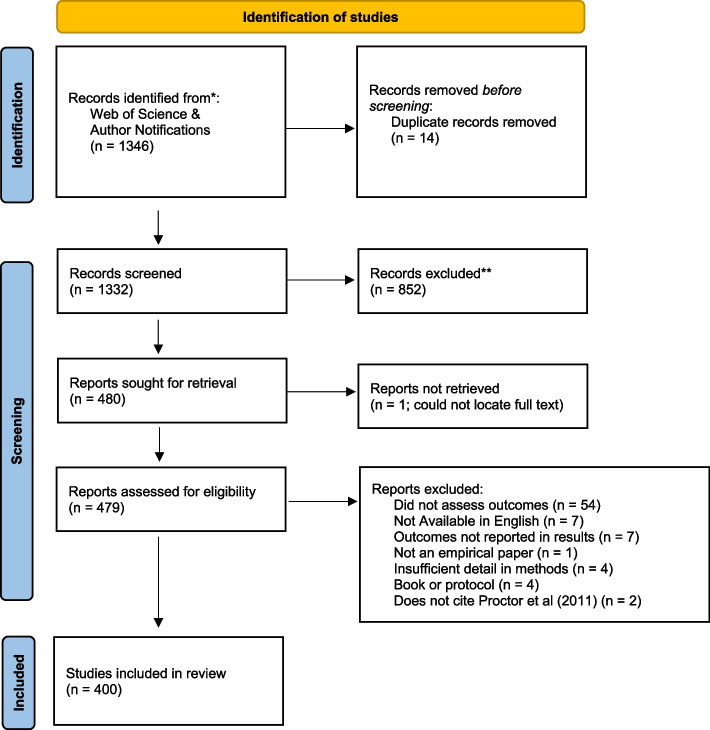
PRISMA diagram

### Impact of the 2011 implementation outcomes paper

The 2011 paper spurred several critical developments in implementation science. Research funding announcements began to note the taxonomy’s importance for study conceptualization and design, including the U.S. National Institute of Health’s PAR 22–105 for Dissemination and Implementation Science in Health which identified these implementation outcomes as important for inclusion in investigator-initiated research applications [2]. Eighteen institutes and centers signed onto this crosscutting PAR.

The Implementation Outcomes Framework joined the ever-growing list of implementation research frameworks [3, 4], with unique contributions. First, the taxonomy signaled to researchers, policymakers, practitioners, and system leaders that implementation science has distinctive, legitimate outcomes warranting study alongside the outcomes traditionally prioritized in intervention trials. Second, the taxonomy provided direction for treating implementation outcomes as key targets of change, spurring the testing of implementation strategies designed to improve this new outcomes category. Third, the taxonomy raised widespread awareness around the lack of tools, instruments, and designs (e.g., hybrids II and III [5, 6]) that support the measurement of implementation outcomes either as standalone research aims or in conjunction with other outcomes and/or variables capturing contextual determinants.

### The 2011 call for advances in the conceptualization and measurement

The first prong of the 2011 research agenda [1] called for advancing the conceptualization and measurement of implementation outcomes through consistent terminology, a call recently echoed by Murrell et al. [7]. The 2011 paper challenged researchers to report the referent for all implementation outcomes and to specify measurement levels and methods [5]. Subsequently, many scholars have helped refine implementation outcome conceptualization.

For example, Lyon and Bruns [70] distinguished two types of implementation outcomes. They proposed that acceptability, appropriateness, and feasibility comprise *perceptual implementation outcomes*, while adoption, fidelity, and reach/penetration are *behavioral implementation outcomes*. An updated Consolidated Framework for Implementation Research (CFIR) distinguished between *anticipated* (forward-looking) and *actual* (backward-looking) implementation outcomes [8]. An *Implementation Science* editorial indicated that *observable implementation outcomes* such as adoption, fidelity, penetration, and sustainability are of most interest to the journal [9].

Significant measurement advances also occurred in response to the 2011 research agenda. The Grid-Enabled Measures Project [10] and The Society for Implementation Research Collaborative (SIRC) Instrument Review Project [11] are organized around the Proctor 2011 taxonomy. Weiner and colleagues’ study of the psychometric properties for measures of three key implementation outcomes [12]. Moullin and colleagues further refined pragmatic measurement via the PRESS measure for provider-rated sustainment in inner contexts [13], while the NoMAD instrument, based on normalization process theory, may also enhance implementation outcomes’ measurement [14]. We now have systematic reviews of implementation outcomes [15] and their measurement properties in behavioral health [16], public policy [17], stroke care [7], and physical healthcare [18]. Given these advancements in measurement tools, the field needs to examine commensurate progress toward their conceptual precision and linguistic harmony.

### The 2011 call for theory building

Improved conceptualization and measurement positions researchers to move from asking descriptive questions about implementation outcomes to causal mechanistic ones [9], which is essential for building testable theory that describes, explains, and predicts how and why the implementation process worked (or not). Accordingly, the second prong of the 2011 research agenda called for theory-building research focused on employing implementation outcomes as key constructs in efforts to model successful implementation [1]. Researchers were challenged to explore the salience of implementation outcomes to different stakeholders and to investigate the importance of various implementation outcomes by phase in the implementation process—both of which can help researchers detect modifiable indicators of successful implementation [1].

Proctor and colleagues also called for research that tests and models various roles that implementation outcomes can play and research that illuminates how different implementation outcomes are associated with one another [1]. Their paper called for researchers to test several types of hypotheses related to how implementation outcomes are associated with each other, how the attainment of implementation outcomes influences service system and clinical outcomes, and how the effectiveness of implementation strategies affects implementation outcome attainment [20]. This call for hypothesis testing in implementation outcomes research has been echoed by a number of recent papers [21–24]. Current literature also reflects an increasing number of studies testing the effectiveness of implementation strategies and the mechanisms that explain how these strategies may influence implementation outcomes [25–31]. A 2021 scoping review paper [7] of adult stroke rehabilitation research using the Proctor 2011 framework revealed that adoption was the most frequently measured implementation outcome. No studies examined implementation cost, and fewer than half found that implementation strategies were effective in attaining implementation outcomes [7].

The 2011 paper also noted that measuring and empirically testing implementation outcomes can help specify the mechanisms and causal relationships within implementation processes and advance an evidence base around successful implementation. Since then, the field has responded. Recent publications raise awareness of mechanisms and advance their conceptualization in the context of implementation research. Emerging topics include prospectively building mechanism-focused hypotheses into research designs, developing approaches for identifying and prioritizing mechanisms, and advancing mechanisms measurement [19, 27]. Overall, the field still lacks conclusive evidence about interrelationships, particularly causal relationships, among implementation outcomes, strategies, subsequent outcomes, and their contextual and strategy determinants.

### Study purpose

This review was designed to examine advances in (1) conceptualization of implementation outcomes (including the outcomes that have received empirical attention, the contexts for their study, and methods employed) and (2) theory building around implementation outcomes (interrelationships among implementation outcomes and their relationship to implementation strategies). We synthesize progress against the challenges posted in the 2011 paper and propose directions for the next 10 years of implementation outcomes research.

## Methods

The first five steps of Arksey and O’Malley’s methodological framework for conducting scoping reviews guided our approach [32]. We also replicated the iterative and reflexive approach modeled by Marchand et al. [33] and Kim et al. [34] during each step of our scoping review process. Our published review protocol describes methods [35]. Here, we summarize and review key steps and note refinements to the protocol.

### Stage 1: Defining the research questions

This review addressed three questions about advances in implementation outcomes conceptualization and measurement:

1. To what extent has each of the eight implementation outcomes been examined empirically in the literature? What other implementation outcomes did these studies identify?
2. What research designs and methods have been used to study each outcome?
3. In what contexts have implementation outcomes been studied? What service settings, populations, health conditions, and innovation types are represented?

To understand advances in theory-building around implementation outcomes, we addressed two additional questions:

4. Which implementation outcomes have been studied as dependent variables in tests of implementation strategy effectiveness?
5. What interrelationships between implementation outcomes have been studied empirically? This includes relationships among implementation outcomes and other outcome types, specifically service, and clinical outcomes.

### Stage 2: Identifying relevant literature

Using forward citation tracking, we identified all literature that cited the 2011 paper and was published between October 2010 (date of online publication) and October 30, 2020. We conducted our search in the WOS database in July 2020. To account for any delays in archiving more recent publications in WOS, we also located articles using citation alerts sent to the first author from the publisher for a 6-month period coinciding with the end of the WOS citation search (February to July 2020). In May 2023, we used the same forward citation tracking procedures in WOS to confirm all articles that cited the 2011 paper and were published through October 2020 because of archiving lags and to collect a full 10 years of implementation outcomes papers. Citations were managed in Mendeley and then exported to Covidence.

### Stage 3: Article selection

As reported in our protocol paper [35], we screened articles and included them if they (a) reported results of an empirical study, (b) were published in a peer-reviewed journal, and (c) were designed to assess at least one of the identified implementation outcomes or their synonyms as specified in the original implementation outcome taxonomy.

### Stage 4: Data charting

Data were charted using a customized Google Form, depicted in Table 1 of the study protocol paper [35]. Since protocol publication, we added two variables: health condition, which was defined as the primary health, disease, or problem targeted by the intervention or prevention effort, and funding source variable, defined as the first listed funder of the study.

**Table 1:** Number and percent of studies by funding source and regional setting (*n* = 400)

	**Number**	**%**
**Funding**		
National Institutes of Health (US)	99	24.5%
National Institute of Mental Health	53	13.3%
National Institute on Drug Abuse	21	5.3%
National Cancer Institute	14	3.5%
National Heart, Lung, and Blood Institute	5	1.3%
National Institute of Child Health & Human Development	6	1.5%
Non-US	103	25.8%
Other US Federal	66	16.5%
None noted/specified	59	14.8%
Other Foundation	44	11.0%
US Veterans’ Administration	9	2.3%
Agency for Healthcare Research and Quality (US)	9	2.3%
US State Funding	5	1.3%
Patient-Centered Outcomes Research Institute (US)	3	0.8%
Industry	2	0.5%
**Region**		
Africa	40	10.0%
Asia	17	4.3%
Australia	18	4.5%
Canada	26	6.5%
Caribbean	3	0.8%
Central America	7	1.8%
Europe	65	16.3%
Middle East	1	0.3%
Not specified	3	0.8%
South America	6	1.5%
USA	227	56.8%

Because studies could be funded by multiple sources, these categories are not mutually exclusive

### Stage 5: Collating, summarizing, and reporting the results

We calculated and report frequencies, averages, and trends over time to identify the extent to which implementation outcomes are studied empirically in the 400 included manuscripts. To identify progress in research on implementation outcomes, we examined the role of implementation outcomes in analyses—as correlates of contextual factors and other implementation outcomes, and as dependent variables in relation to implementation outcomes. 

## Results

**Fig. 2:**
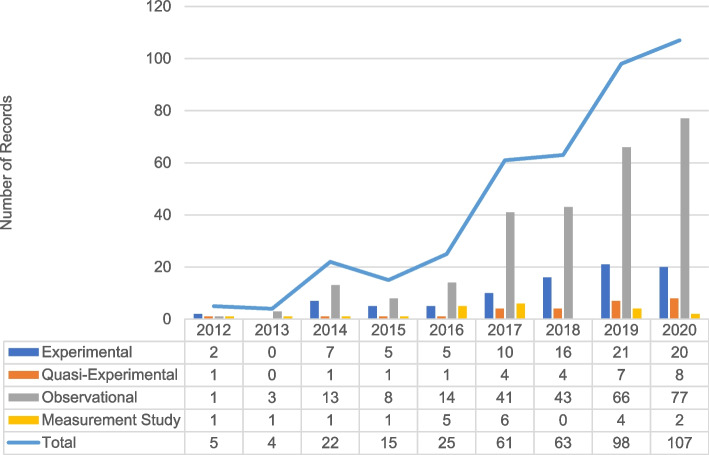
Number of included records and study types by year of publication (*n* = 400)

Our identification process generated 1346 abstracts for screening, which yielded 479 manuscripts for full-text review. After a full-text review, we excluded 79 manuscripts. A total of 400 manuscripts met the inclusion criteria (Fig. 1). Among the manuscripts qualifying for a full-text review, 82% were published in or after 2017 (Fig. 2). A wide range of funders supported implementation outcomes research globally and domestically. The National Institutes of Health (NIH)—especially the National Institute for Mental Health (NIMH)—was the most frequent funding source (24.5%). We found little evidence of foundation, state, or the Patient-Centered Outcomes Research Institute (PCORI) funding (Table 1).

### Question 1: To what extent has each of the eight implementation outcomes been examined empirically in the literature? What additional implementation outcomes were identified?

More than half (52%) of the included manuscripts examined acceptability, followed by fidelity (38.8%), feasibility (36.9%), adoption (24.0%), and appropriateness (20.1%). Penetration (15.4%), sustainability (15.1%), and cost (7.5%) were examined less frequently (Table 2). Most manuscripts indicated the stage or phase of implementation investigated, which we coded using the EPIS framework (exploration, adoption/preparation, implementation, sustainment). Focus on implementation outcomes varied by stage or phase, bearing out projections in the 2011 paper. In studies conducted during the exploration phase, appropriateness, feasibility, acceptability, and adoption were most frequently examined. Adoption, cost, and feasibility were addressed most frequently in studies conducted during the preparation phase. As hypothesized in 2011, sustainability was the outcome examined most during sustainment phase studies.

**Table 2:** Coverage of implementation outcomes (*n* = 400)

	All (*n* = 400)	Acceptability	Adoption	Appropriateness	Cost	Feasibility	Fidelity	Penetration	Sustainability
		(*n* = 210)	(*n* = 106)	(*n* = 87)	(*n* = 31)	(*n* = 154)	(*n* = 157)	(*n* = 64)	(*n* = 63)
***% of included studies***	100.0%	52.1%	26.5%	21.8%	7.8%	38.6%	39.3%	16.0%	15.8%
***Implementation phase***									
Exploration	9.5%	11.4%	9.4%	13.8%	12.9%	13.6%	7.0%	9.4%	6.3%
Preparation	20.5%	19.5%	35.8%	17.2%	29.0%	18.8%	14.6%	26.6%	20.6%
Implementation	64.5%	67.6%	61.3%	62.1%	83.9%	66.9%	75.2%	75.0%	69.8%
Sustainment	11.3%	6.7%	15.1%	3.4%	16.1%	7.8%	15.3%	18.8%	44.4%
Not specified	8.5%	10.5%	10.4%	11.5%	0.0%	9.7%	7.0%	4.7%	1.6%

Eight percent (*n* = 32) of manuscripts identified implementation outcomes that were not in the original taxonomy. Our coding’s free text entry captured 24 unique alternative implementation outcome constructs, including *evidence of delivery* (e.g., use, provision, or receipt of an intervention; *n* = 4), *usefulness* (e.g., usability, utility; *n* = 14), clients’ *responsiveness/engagement* (*n* = 4), features of the intervention (e.g., adaptability, effectiveness; *n* = 7); clinician features (e.g., efficacy, competence; *n* = 8), *level of implementation* (*n* = 1), *scale up* (*n* = 1), and *timely initiation* (*n* = 1). Some of these terms (e.g., provider skill) may reflect determinants of implementation. Others—notably usefulness, usability, and utility—were identified in the 2011 paper as “other terms in the literature.”

### Question 2: What research designs and methods have been used to study each outcome?

As Table 3 shows, most analyses of implementation outcomes were descriptive, with two-thirds employing on observational designs (*n* = 266). Experimental (*n* = 86, 21.5%) and quasi-experimental studies (*n* = 27; 6.8%) were less common; these studies accounted for about 30% of manuscripts every year, and this proportion did not fluctuate greatly over time (Fig. 2). Acceptability, adoption, and fidelity were most likely to be studied through experimental designs. Appropriateness was most likely to be studied qualitatively. Quantitative methods were used primarily for assessing adoption, cost, fidelity, and penetration. Less than a third of manuscripts presented mixed or multiple methods.

**Table 3:** Design and methodological approaches identified in implementation outcomes research (*n* = 400)

	All	Acceptability	Adoption	Appropriateness	Cost	Feasibility	Fidelity	Penetration	Sustainability
	(*n* = 400)	(*n* = 210)	(*n* = 106)	(*n* = 87)	(*n* = 31)	(*n* = 154)	(*n* = 157)	(*n* = 64)	(*n* = 63)
***Design***									
Instrument development	5.3%	2.9%	3.8%	4.6%	3.2%	4.5%	6.4%	1.6%	1.6%
Observational	66.5%	70.0%	67.0%	71.3%	67.7%	73.4%	58.0%	76.6%	82.5%
Quasi-experimental	6.8%	7.1%	9.4%	8.0%	6.5%	6.5%	6.4%	7.8%	14.3%
Experimental	21.5%	20.0%	19.8%	16.1%	22.6%	15.6%	29.3%	14.1%	1.6%
***Number of observations***									
Cross-sectional	42.8%	45.2%	40.6%	47.1%	38.7%	42.2%	29.3%	43.8%	44.4%
Pre-post	15.3%	22.4%	17.0%	17.2%	12.9%	21.4%	16.6%	12.5%	12.7%
Longitudinal (> 3)	36.5%	29.0%	38.7%	29.9%	45.2%	31.2%	47.8%	42.2%	41.3%
***Type of data used to assess***									
Qualitative	17.5%	33.3%	19.8%	44.8%	19.4%	23.4%	13.4%	12.5%	28.6%
Quantitative	48.5%	37.1%	66.0%	24.1%	67.7%	48.1%	66.2%	68.8%	44.4%
Multi/mixed	32.5%	28.6%	12.3%	29.9%	12.9%	27.9%	17.2%	17.2%	23.8%
***Role in analysis***									
Descriptive (e.g., univariate)	62.5%	87.6%	78.3%	92.0%	87.1%	87.0%	68.8%	76.6%	79.4%
Correlation (e.g., association)	8.3%	6.2%	12.3%	4.6%	3.2%	5.2%	12.7%	14.1%	19.0%
Independent variable (e.g., predictor)	4.5%	3.8%	3.8%	2.3%	3.2%	1.3%	9.6%	6.3%	3.2%
Dependent variable (e.g., outcome)	24.3%	11.0%	19.8%	8.0%	12.9%	11.7%	29.3%	23.4%	22.2%

### Question 3: In what contexts have implementation outcomes been studied? What service settings, populations, health conditions, and innovation types are represented in the studies?

To describe the context in which implementation outcomes have been studied, we captured study settings and populations, the innovations (implementation objects [36]) studied, and the health conditions addressed by the study (Table 4). Most manuscripts were situated in healthcare (*n* = 183, 45.8%) or behavioral health (*n* = 90, 22.5%) organizations—both inpatient and outpatient, with an additional 50 manuscripts (12.5%) set in schools. Studies predominantly addressed mental health (*n* = 129, 32.3%) or medical (*n* = 103, 25.8%) concerns. Manuscripts varied in their focus on the age group, with some including more than one age group. Nearly two-thirds of studies addressed adults and over 40% included children. The most common implementation object studied was a single evidence-based practice (*n* = 161, 40.3%). Implementation outcomes were studied in relation to screening and technological innovations in fewer than 22% of the manuscripts.

**Table 4:** Service context features in implementation outcomes research (*n* = 400)

	ALL	Acceptable	Adoption	Appropriate	Cost	Feasibility	Fidelity	Penetration	Sustainability
	(*n* = 400)	(*n* = 210)	(*n* = 106)	(*n* = 87)	(*n* = 31)	(*n* = 154)	(*n* = 157)	(*n* = 64)	(*n* = 63)
***Setting***									
Healthcare	45.8%	51.0%	64.2%	51.7%	41.9%	49.4%	38.9%	57.8%	50.8%
Behavioral health	22.5%	19.0%	14.2%	23.0%	35.5%	19.5%	24.8%	15.6%	22.2%
School	12.5%	9.5%	7.5%	6.9%	9.7%	8.4%	18.5%	4.7%	6.3%
Social service	6.3%	4.8%	2.8%	6.9%	3.2%	5.8%	5.1%	4.7%	4.8%
Other community based	5.5%	6.7%	3.8%	10.3%	3.2%	7.8%	5.7%	6.3%	7.9%
Child welfare	3.0%	2.4%	4.7%	2.3%	3.2%	1.9%	1.3%	1.6%	1.6%
Not specified	2.5%	2.9%	1.9%	0.0%	0.0%	2.6%	3.2%	3.1%	3.2%
Universities	2.5%	3.3%	1.9%	0.0%	0.0%	2.6%	1.9%	0.0%	0.0%
Corrections/law enforcement	2.3%	2.9%	0.9%	0.0%	3.2%	3.9%	3.2%	1.6%	4.8%
Public health	1.5%	1.9%	2.8%	2.3%	0.0%	1.3%	0.6%	1.6%	0.0%
Other	1.8%	1.9%	0.9%	1.1%	3.2%	1.3%	1.3%	1.6%	1.6%
***Population***									
Adult	61.5%	66.7%	66.0%	66.7%	51.6%	65.6%	58.6%	65.6%	60.3%
Children	41.5%	32.9%	38.7%	32.2%	38.7%	35.1%	52.2%	39.1%	39.7%
Older adult	8.3%	9.5%	9.4%	9.2%	3.2%	6.5%	7.0%	14.1%	9.5%
Vulnerable population	5.5%	6.7%	0.9%	5.7%	3.2%	10.4%	2.5%	3.1%	4.8%
Not specified	3.0%	3.8%	4.7%	4.6%	12.9%	3.2%	1.3%	1.6%	4.8%
Parents/families	2.0%	2.9%	0.0%	2.3%	0.0%	2.6%	2.5%	0.0%	0.0%
Practitioners	1.5%	1.4%	0.0%	0.0%	0.0%	1.9%	1.9%	0.0%	0.0%
***Innovation***									
Single EBP (one manualized treatment or program)	40.3%	39.5%	22.6%	39.1%	45.2%	40.9%	51.6%	28.1%	28.6%
Screening, assessment, or diagnostic procedure (e.g., X-rays)	13.0%	14.3%	15.1%	13.8%	3.2%	13.0%	10.2%	17.2%	11.1%
Technology (health information technology, health app)	8.8%	11.0%	14.2%	10.3%	12.9%	11.0%	5.7%	7.8%	14.3%
Other	8.0%	9.5%	9.4%	9.2%	3.2%	8.4%	0.6%	9.4%	9.5%
Multiple EBPs	7.3%	5.2%	7.5%	5.7%	6.5%	4.5%	7.6%	12.5%	12.7%
Implementation strategy (e.g. learning collaborative)	7.5%	6.2%	13.2%	4.6%	0.0%	5.8%	10.2%	9.4%	9.5%
Clinical pathway or service cascade (screening, referral, treatment)	7.5%	7.1%	12.3%	6.9%	9.7%	10.4%	7.0%	7.8%	7.9%
Research evidence (in general)	5.8%	3.8%	8.5%	2.3%	9.7%	4.5%	5.1%	9.4%	9.5%
Guideline	5.0%	3.3%	8.5%	3.4%	6.5%	4.5%	4.5%	9.4%	6.3%
Administrative (e.g., billing system, supervision approach, marketing)	5.0%	4.8%	6.6%	3.4%	9.7%	3.9%	2.5%	3.1%	3.2%
Outcomes monitoring (e.g., measurement-based care)	2.5%	3.8%	1.9%	4.6%	3.2%	3.9%	1.9%	3.1%	0.0%
Data system (indicators or monitoring systems)	2.0%	1.9%	3.8%	1.1%	3.2%	1.9%	1.9%	0.0%	3.2%
Policy	0.8%	1.0%	0.0%	0.0%	0.0%	0.0%	0.6%	0.0%	1.6%
Not indicated	0.5%	0.0%	0.0%	1.1%	0.0%	0.0%	0.6%	0.0%	0.0%
***Health condition***									
Substance use (inc. tobacco)	8.0%	8.1%	6.6%	6.9%	12.9%	5.8%	4.5%	10.9%	9.5%
Mental health	32.3%	29.5%	20.8%	27.6%	29.0%	32.5%	35.0%	23.4%	31.7%
Cancer	5.0%	4.8%	5.7%	6.9%	3.2%	5.8%	2.5%	7.8%	4.8%
General medical	25.8%	24.3%	37.7%	33.3%	32.3%	29.2%	27.4%	32.8%	30.2%
HIV/AIDS	6.3%	7.1%	5.7%	4.6%	0.0%	5.8%	5.7%	7.8%	6.3%
Infectious disease (non-HIV)	3.5%	3.3%	3.8%	3.4%	6.5%	3.2%	3.8%	1.6%	3.2%
Neuro-cognitive disorders (e.g., dementia)	2.0%	2.4%	0.9%	1.1%	0.0%	2.6%	1.9%	1.6%	1.6%
Reproductive/antenatal care	2.8%	4.3%	6.6%	4.6%	3.2%	2.6%	3.8%	4.7%	3.2%
Communication disorders	0.8%	1.4%	0.9%	0.0%	0.0%	0.6%	0.6%	0.0%	0.0%
Academic achievement	1.5%	2.9%	0.9%	0.0%	0.0%	1.3%	1.3%	1.6%	0.0%
Maltreatment/injury/violence	5.8%	6.2%	4.7%	6.9%	6.5%	6.5%	5.1%	3.1%	3.2%
Employment/economic well-being	0.0%	0.0%	0.0%	0.0%	0.0%	0.0%	0.0%	0.0%	0.0%
Workforce/administration	2.5%	2.4%	3.8%	2.3%	3.2%	0.6%	1.3%	4.7%	3.2%
Developmental disabilities	4.0%	3.3%	1.9%	2.3%	3.2%	3.2%	7.0%	0.0%	3.2%

### Question 4: Which outcomes have been studied as dependent variables in tests of implementation strategy effectiveness—a theory-building question?

**Fig. 3:**
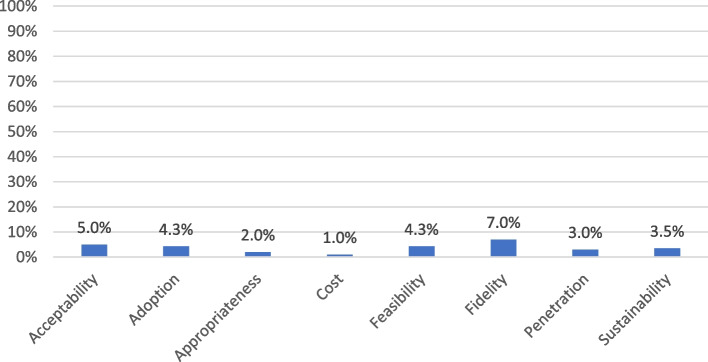
Percentage of included records that examined implementation strategies, by implementation outcome (*n* = 400)

**Fig. 4:**
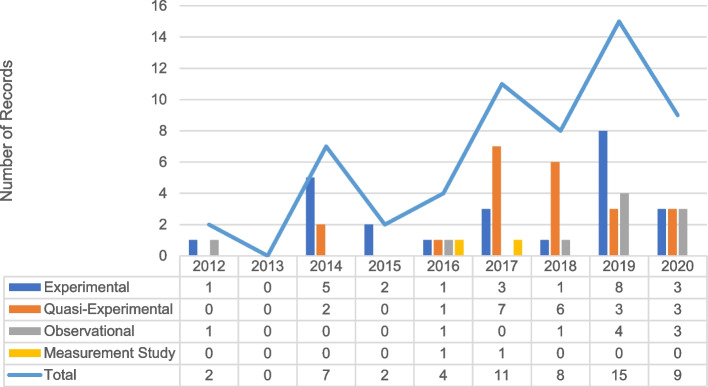
Designs used to examine implementation strategies and outcomes over time (*n* = 56)

Despite being conceptualized as outcomes (because of exposure to different conditions and strategies), implementation outcomes were treated as dependent variables in only one-quarter (*n* = 97) of included manuscripts. Only 56 (14.0%) manuscripts examined implementation outcomes in relation to implementation strategies. Fidelity was most frequently studied as an outcome of implementation strategies (7.0%) (Fig. 3). Although over half of the manuscripts examined acceptability, only 5.0% assessed its role as an outcome of implementation strategies. Similarly, few manuscripts presented tests of implementation strategies for their ability to attain fidelity, feasibility, appropriateness, or address cost barriers. Most manuscripts examining implementation strategies presented experimental (*n* = 24) or quasi-experimental (*n* = 22) designs (Fig. 4).

### Question 5: What interrelationships between implementation outcomes have been studied empirically? This theory-building question includes relationships among implementation outcomes and other outcome types, specifically service and clinical outcomes

Finally, we examined the role of each implementation outcome in the analysis (Tables 5 and 6). Fifteen percent of included manuscripts examined relationships between implementation outcomes and other outcomes. Only 5.0% (*n* = 21) tested relationships among different implementation outcomes. As Tables 5 and 6 show, the cost was not examined in relation to other implementation outcomes. Sustainability was examined most often, particularly in relation to fidelity (*n* = 3), penetration (*n* = 3), and adoption (*n* = 2).

**Table 5:** Percentage of studies that examined implementation outcomes relative to other outcomes (by implementation outcome) (*n* = 400)

	All	Acceptability	Adoption	Appropriateness	Cost	Feasibility	Fidelity	Penetration	Sustainability
	(*n* = 400)	(*n* = 210)	(*n* = 106)	(*n* = 87)	(*n* = 31)	(*n* = 154)	(*n* = 157)	(*n* = 64)	(*n* = 63)
Any	15.0%	8.6%	14.2%	4.6%	16.1%	9.1%	19.7%	17.2%	17.5%
Implementation	5.3%	3.3%	8.5%	2.3%	0.0%	2.6%	5.7%	6.3%	11.1%
Service system	4.8%	1.9%	3.8%	0.0%	16.1%	4.5%	4.5%	4.7%	6.3%
Client	5.5%	3.3%	1.9%	2.3%	0.0%	2.6%	10.2%	7.8%	1.6%

**Table 6:** Number of studies that examined interrelationships among implementation outcomes (*n* = 21)

	Acceptability	Adoption	Appropriateness	Cost	Feasibility	Fidelity	Penetration	Sustainability
Acceptability	-	3	1	0	3	2	0	0
Adoption		-	0	0	1	1	1	2
Appropriateness			-	0	1	1	1	1
Cost				-	0	0	0	0
Feasibility					-	0	0	1
Fidelity						-	3	3
Penetration							-	3
Sustainability								-

As shown in Table 7, only 23 manuscripts (5.8%) examined implementation outcomes in relation to service outcomes. Among implementation outcomes, feasibility (*n* = 9) was most often correlated with service outcomes. Effectiveness (*n* = 15) was the service outcome most frequently tested in relation to implementation outcomes. No studies of implementation outcomes in our sample addressed service outcomes of safety or equity. We also coded whether each manuscript examined implementation outcomes in relation to clinical outcomes, although given the wide heterogeneity in clinical outcomes of interest and in the absence of a corresponding taxonomy, we did not categorize specific clinical outcomes in this review. Only 22 studies (5.5%) examined implementation outcomes in relation to clinical outcomes. Fidelity was the implementation outcome most examined relative to clinical outcomes (10.2% of the manuscripts).

**Table 7:** Implementation outcomes and service system outcomes (*n* = 22)

	Service system outcomes						**Total**
	Efficiency	Safety	Effectiveness	Equity	Patient-centeredness	Timeliness	
Implementation outcomes							
Acceptability	0	0	2 –	0	1	0	3
Adoption	1 *	0	1 *	0	0	1 *	3
Appropriateness	0	0	0	0	0	0	0
Cost	1	0	2	0	0	0	3
Fidelity	0	0	2 *	0	0	0	2
Feasibility	0	0	7 * -*	0	1	1	9
Penetration	0	0	0	0	0	1 *	1
Sustainability	0	0	1	0	0	0	1
**Total**	2	0	15	0	2	3	22

Not all studies reporting findings indicated the direction of relationships

^*^Significant positive relationship reported

^−^Null effect reported

## Discussion

One decade later, this scoping review assessed the field’s response to the 2011 paper’s research agenda calling for advances in conceptualization, measurement, and theory-building around implementation outcomes. Our results show a proliferation of literature on implementation outcomes. However, empirical investigations accounted for less than one-third of manuscripts citing the 2011 paper. While descriptive work can enrich our conceptual understanding of implementation outcomes, more work remains to advance a theory that explains the attainment and effects of implementation outcomes.

### How has research on implementation outcomes advanced over the past 10 years?

Implementation outcomes research is supported by a range of funding sources and is conducted in many settings and disciplines. Most included studies were conducted in health and behavioral health organizations. Similar research is needed in less frequently studied settings where health and other social care interventions are delivered (e.g., schools, social service organizations, and home-based services) [37–42] to diverse communities and consumers with a range of intersecting needs. The context for implementation, often varying by setting, has been shown to affect certain implementation outcomes [43]. Building knowledge in varying settings can help advance conceptualization and theory building around implementation outcomes like penetration (or reach), propel incorporation of equity in the study of implementation outcomes, and provide unique opportunities to further articulate the relationships between implementation outcomes and other service outcomes, particularly equity.

Most included studies examined the implementation of a single evidence-based intervention or implementation object, failing to capture the reality of organizations and systems that typically work to introduce, implement and sustain the simultaneous delivery of multiple interventions. Studying the implementation of multiple interventions carries logistic, resource, and design challenges but can make scientific leaps, particularly regarding external validity. Future research should examine how service system directors weigh acceptability, feasibility, and cost while selecting interventions and strategies and how they juggle simultaneous implementation efforts, stagger their timing, and sustain them in dynamic and unpredictable environments.

Our results reflected considerable variation in the degree to which different implementation outcomes have been studied, with a heavy emphasis on acceptability, echoing other recent reports. In a systematic review of quantitative measures assessing health policy implementation determinants and outcomes, Allen and colleagues found that acceptability, feasibility, appropriateness, and compliance were most frequently measured [17]. Moreover, Mettert and colleagues reported that acceptability had the greatest number of measurement options [15]. Other implementation outcomes like cost, penetration, and sustainability (the observable implementation outcomes prioritized by *Implementation Science* [9]) were measured less frequently in our review sample.

This suggests that, currently, implementation outcomes research reveals more about which interventions and strategies people like (important for refining interventions, improving patient-centeredness, and supporting initial uptake), but less about the degree to which interventions reach and benefit communities. Insufficient attention to outcomes like penetration and cost (those highly prioritized in real-world decision making) limits our field’s ability to take evidence-based practices to scale for public health impact. Building strong evidence about these more observable implementation outcomes is critical for supporting policymakers and program leaders as they make decisions about strategic priorities and resource allocation to deploy, scale, and sustain interventions that will reach an adequate number of consumers equitably.

Our review explored the field’s progress toward conceptual and linguistic harmony and the promise of uncovering new implementation outcomes. Some manuscripts cited the 2011 paper but employed alternative concepts and terminology for implementation outcomes despite their close alignment with the 2011 taxonomy. For example, terms such as “evidence of delivery,” “use,” “provision,” or “receipt of services” could be more precisely operationalized by adoption or penetration. Similarly, outcomes such as “client response,” “participant responsiveness,” and “engagement” align closely with the term acceptability. Where authors discover granular distinctions between more commonly used terms, a rationale for proposing new terms is welcome and necessary. Nonetheless, we reiterate the importance of common implementation outcome terminology, where possible, so that the field can continue to build and harmonize knowledge across studies. Moreover, some of the alternative terms may be more accurately labeled as determinants of implementation outcomes rather than new outcomes (e.g., client and provider factors).

The results of our review also identified emerging implementation outcomes that are distinct from those proposed in the 2011 taxonomy. For example, there has been widespread attention to scale-up [44–49]. Although the 2011 paper conceptualized actual or perceived utility as a synonym for feasibility and usefulness as a synonym for appropriateness, the number of studies using this term as a distinct outcome suggests that perceived usefulness, usability, and utility may be conceptually distinct from constructs in the 2011 outcome taxonomy. The expansion of implementation outcomes taxonomy was encouraged by Proctor et al. in the 2011 manuscript. For such outcomes, we encourage the provision of common use and operational definitions, psychometric research to refine measurement, and clear reporting and justification for how these are conceptually distinct from the original taxonomy.

Reflecting the phased nature of implementation, Proctor et al. 2011 proposed that some implementation outcomes might be most salient—and thus likely to be measured—at different times [1]. Although all outcomes were likely to be studied during active implementation phases, outcomes like appropriateness, feasibility, acceptability, and adoption were especially common in studies conducted during the early phases of exploration and preparation. Outcomes like cost, fidelity, penetration, and sustainability were more common during later implementation and sustainment phases. This may reflect the importance of different implementation outcomes for decision making over time and at certain points in the implementation lifecycle. However, we found little evidence of testing hypotheses about the optimal order of attaining specific implementation outcomes. We hope this can be improved as methods such as causal pathway diagrams, causal loop diagrams, and directed acyclic graphs gain traction in mechanistic implementation research [19, 30, 50–53].

### More theory-building work and more experimental studies are needed

Our results suggested limited progress toward theory development. Few manuscripts focused on explaining, testing, or modeling the processes that reveal how implementation outcomes can change, be intervened upon, or affect other outcomes. Few studies treated implementation outcomes as dependent variables in studies that investigate associations or causal relationships between determinants and implementation outcomes. We also found few studies testing the relationships between implementation strategies and implementation outcomes—a key part of the 2011 proposed agenda. This gap is concerning given the purpose of implementation science, that is, to advance strategies for integrating innovations into everyday practice. Our results suggested that implementation scholars are still in the early stages of building evidence for the causal effects of implementation determinants and strategies and still do not know how to achieve implementation outcomes. We hope that can be ameliorated with a continued increase in study designs that include prospective theorizing about what mechanisms explain strategy effectiveness and precise measurement of these mechanisms in relation to specific implementation outcome attainment [19, 27, 54].

Although some have questioned testing implementation outcomes as dependent variables [55], rigorous trials of implementation strategies are important for learning how to achieve acceptability, feasibility, adoption, and sustainment. For example, random assignment of train-the-trainer or coaching to clinics can inform the most effective approach to provider adoption. Debate also surrounds the question of whether or not implementation outcomes are ever sufficient as “endpoint”-dependent variables and whether they should always be tested in relation to more distal service systems and clinical outcomes (as discussed below). While we argue for more research testing the intermediate role of implementation outcomes, testing their role as endpoint-dependent variables seems warranted as we continue to advance knowledge about how to most effectively attain them, and which implementation strategies to prioritize and invest in to do so.

Though correlational studies serve the function of suggesting variables for further testing to reveal building blocks for theory, scientific leaps require a shift from the descriptive work that, as evidenced by our findings, dominates the field. Though observational research is important for laying a foundation, particularly as implementation research moves into newer fields and settings (e.g., large-scale policy implementation), theoretical advances are necessary to understand how contextual factors such as organizational leadership [24] and implementation strategies affect outcome attainment. More work is needed to specify and test mechanistic pathways and empirical hypotheses about drivers, moderators, and mediators of implementation outcomes in a replicable way so that it is clear what knowledge is generalizable across settings versus what needs to be learned and assessed locally. Furthermore, finer-grained identification of the measurable proximal outcomes that precede implementation outcome attainment can help us better understand how exactly a strategy works to improve the implementation outcome(s) it is targeted to change (and thus what is core vs. adaptable about the strategy itself), as well as more clearly isolate what factors are not addressed by the strategy and thus need additional attention in order to achieve the desired implementation outcome(s). Notably, the frequency with which mixed methods were employed in our sample suggested the availability of rich data to pursue the theoretical advances we encourage here.

Studies in our reviews rarely addressed relationships among implementation outcomes. Given our finding that various implementation outcomes might be more salient at different phases, studies should examine the optimal temporal ordering of their pursuit. For instance, clinician perceptions about the acceptability, appropriateness, and feasibility of an intervention might predict adoption [56]. Longitudinal studies that measure and test relationships among multiple implementation outcomes before, during, and after implementation can generate new insights about phasing implementation efforts and the potential additive and interactive effects of thoughtful sequencing.

Few studies tested hypothesized impacts of implementation outcomes on other important outcome types, such as service system changes and improved individual or population health, thereby limiting theory building and testing the impact of implementation outcomes. This finding echoes recent reflections on the promises and pitfalls of implementation science [54] and suggests that our field has yet to empirically demonstrate the value of implementation science for improving health and healthcare quality.

Such inquiry is critical in work to reduce health disparities [40, 57–61]. Equity is a key service system outcome [1, 62]. Delivering interventions that are unacceptable to clients will surely block equitable care. Data on acceptability and feasibility can be used to adapt interventions and the associated implementation processes to build local capacity. Using implementation outcomes, equity in service delivery may be modeled and tested as follows:

$$\mathrm{Equity }= f\mathrm{\, of\, service\, acceptability }+\mathrm{ feasibility }+\mathrm{ appropriateness}$$

Similarly, penetration and sustainment of evidence-based care to the entirety of a system’s service recipients or a community’s under-resourced populations can serve as measurable indicators of equitable access and reach [63, 64], consistent with calls to prioritize structural racism in contextual analyses [65]. We hypothesize the following:

$$\text{Equitable access }= f\ \text{of fidelity} + \mathrm{ penetration} + \text{sustainment of evidence}-\text{based care}$$

$$\mathrm{Adoption }= f\mathrm{\, of\, feasibility\, and\, appropriateness}$$

[63–65]. Future studies that investigate relationships among different outcome types are necessary for achieving the system and population health impact that motivates the field of implementation science and are essential for demonstrating tangible impact and the value of investing in implementation work.

### Strengths and limitations of our review

Our paper complies with recommendations that review articles in implementation science be rigorous, comprehensive of the questions being asked, and provide accurate attributions [66]. Given our review’s aims, we included only articles that cited the 2011 Proctor et. al implementation outcomes paper. Thus, our results likely underestimated advances in the field from studies anchored in alternative theories and taxonomies (e.g., those anchored by the RE-AIM framework), particularly those in adjacent disciplines or that focus on alternative implementation outcomes. Our rigorous calibration process to ensure reliability in the screening and data charting phases, and the iterative adaptation of our data charting procedures contributed to the strength of our review. For example, when coding revealed the need for new variables, we re-reviewed all articles. The reviewed articles presented many coding challenges, particularly around the precision of reporting, which could have introduced errors during the data charting. See Lengnick-Hall et al. [67] for detail on the coding challenges we encountered, along with recommendations to improve reporting.

When juxtaposed with Proctor et al.’s 2011 recommendations and a recent paper on recommendations for reporting implementation outcomes [67], our data provide a basis for projecting priorities for a “next stage agenda” on implementation outcomes 2022–2023. Summarized in Table 8, work must further harmonize implementation outcome terminology. Beyond observational measurement of implementation outcomes, studies should specify their role in analyses, test how to achieve them, and demonstrate their impact on clinical, system, and public health improvements. Especially pressing is understanding how implementation outcomes—particularly acceptability, feasibility, and sustainability—can advance equitable health service delivery. Testing hypothesized sequencing, impact, and efficiency of attaining implementation outcomes via strategies is essential to understanding and accelerating implementation processes [68, 69].

**Table 8:** Agenda for implementation outcomes research: 2022–2032

#1 Research on conceptualization and measurement of implementation outcomes			
**Item #**	**2011 agenda**	**Progress evident in this scoping review**	**Priorities/recommendations for 2022 agenda**
1	2011 agenda argued for consistency of terminology	This issue persists. * Most articles used terms from the 2011 taxonomy but some used new and unclear terms	**Use consistent terminology, specify the referent, and report the level of measurement for each implementation outcome**. * Investigators should capture implementation outcome changes over time and speed of their attainment. ** • Does attainment of a given outcome sustain over time? • How long does it take to achieve such outcomes as fidelity, acceptability?
2	2011 agenda stated that *“researchers should report the referent for all implementation outcomes measured”* (pg. 71)	This issue persists. * Coding was challenged by a lack of clarity	
3	2011 agenda drew our attention to the fact that *“Currently, very few studies reporting implementation outcomes specify the level of measurement, nor do they address issues of aggregation within or across levels”* (pg. 71)	This issue persists. * Coding was challenged by limited and confusing reporting of the level of measurement for implementation outcomes Few studies reported clear time frames for observation windows	
4	2011 agenda noted that *“The actual words used by stakeholders may or may not reflect the terms used in academic literature and reflected in our proposed taxonomy (acceptability, appropriateness, feasibility, adoption, fidelity, penetration, sustainability, and costs). But such research can identify the terms and distinctions that are meaningful to implementation stakeholders”* (pgs. 71–72)	Eight percent (*n* = 32) of manuscripts coded implementation outcomes that were not in the original taxonomy. Some of these terms (e.g., provider skill) may reflect determinants of implementation; others—notably usefulness, usability, and utility—were identified in the 2011 paper as “other terms in the literature.”	**Advance the identification of new implementation outcomes** • Identify and define—conceptually and operationally—new implementation outcomes, demonstrating and explaining their distinction from outcomes in existing typologies
5	2011 paper suggested implementation outcomes’ salience by stage but did not offer specific recommendations	Most manuscripts indicated the stage or phase of implementation investigated, which we coded using the EPIS framework. Few reported observation windows	**Capture the time to achieve implementation outcomes** • Define and capture theory-relevant phases • Identify meaningful observation periods; establish and report metrics
6	2011 agenda asserted that “*Measurement development is needed to enhance the portability and usefulness of implementation outcomes in real-world settings of care*” (pg. 72)	More than half (52%) of the included manuscripts examined acceptability, followed by fidelity (38.8%), feasibility (36.9%), adoption (24.0%), and appropriateness (20.1%). Penetration (15.4%), sustainability (15.1%), and cost (7.5%) were examined less frequently (Table 2). This mirrors the difference in availability of measures for different outcome types (explained in our Discussion)	**Advance precision in measurement** • Leverage measurement resources (e.g., SIRC measurement project) • Capture implementation outcomes using a range of sources: e.g., agency records, participant observation, and other ethnographic methods • Report data sources, frequency of measurement, and metrics. * • Perform and report psychometric properties of measures used
7	2011 paper presented the original taxonomy	Most studies in our review used the original terms or synonyms presented in the 2011 paper. Eight percent (*n* = 32) of manuscripts coded identified implementation outcomes that were not in the original taxonomy	**Conduct deeper analyses of conceptual and operational definitions** for each of the eight outcomes, capturing variation and shedding light on a range of measurement approaches
8	Not explicitly mentioned in 2011 agenda	Most papers in scoping review employed observational designs and reported only descriptive statistics. Very few employed experimental designs. Several used mixed methods	**Leverage design innovations in studying implementation outcomes** • Employ optimization approaches and intervention mapping to unpack mechanisms of “getting to implementation outcomes.” • Utilize newly developed research approaches (including rapid ethnography and human/user-centered designs) that can amplify and clarify participant perspectives on salience of implementation outcomes • Employing adaptive designs; test changes in implementation outcomes as a result of adapting evidence-based interventions • Leverage “SMART” designs to enable real-time adaptation or change in strategies employed in response to real-time data about implementation outcome attainment
**#2 Theory-building research**			
**Item #**	**What 2011 agenda said**	**Progress evident in this scoping review**	**Priorities/recommendations for the 2022 agenda**
9	2011 agenda called for the field to: build a theory that includes the identification and testing of change mechanisms; model and test interrelationships among implementation outcomes; examine implementation outcomes in relation to service and clinical outcomes	Most studies in the scoping review used observational approaches. Very few studies employed experimental designs capable of modeling relationships between various implementation outcomes, between implementation strategies and implementation outcomes, or between implementation outcomes and subsequent outcomes (e.g., service, clinical)	**Conduct theory-based and theory-building studies, especially in relation to implementation phases** • Leverage existing toolboxes of frameworks, methods, and measures to facilitate theory-building research • Test and accrue evidence about causal relationships with implementation outcomes (including measurable hypotheses about how and why a strategy works to change an implementation outcome) • Examine the salience of outcomes by phase and time to attainment • Examine interrelationships among implementation outcomes, capturing temporal aspects and dynamic iterative and/or interactive effects • Test the role of implementation outcomes in attaining service outcomes, especially equity (see Item #12)
10	2011 agenda discussed the importance of modeling interrelationships among implementation outcomes	Fifteen percent of included manuscripts examined relationships between implementation outcomes and other outcomes	**Examine the impact of implementation outcomes on service system outcomes** • Empirically test and model relationships, measuring over time and phases
11	2011 agenda discussed the importance of modeling the attainment of implementation outcomes	Implementation outcomes were treated as dependent variables in only one-quarter (n = 97) of included manuscripts	**Examine the effectiveness of implementation strategies for attaining implementation outcomes, recognizing that many routes may lead to their attainment (equifinality)** • Conduct more research to rigorously test theoretically derived predictions of the role of implementation outcomes in mechanisms of change
12	Equity is mentioned only in Fig. 1 in 2011 paper	No articles in the scoping review examined the role of implementation outcomes in relation to equity. The field has scant empirical evidence about how to improve equity in implementation	**Map inequity and equity by including measures of implementation outcomes** • For various subgroups, is the evidence acceptable? Are evidence-based interventions and implementation strategies feasible? Is an evidence-based intervention sustainable in specific contexts (why and why not)? • Examine implementation outcomes as signals or harbingers of inequity • Posit and test various strategies to improve acceptability, feasibility, sustainability • Posit and test interrelationships between implementation outcomes (e.g., feasibility → adoption)

^*^Lengnick-Hall et al. [67]

^**^Proctor et al. [68]

## Conclusion

This review illustrated growth in implementation outcomes research, but with empirical evaluation reflecting a small subset of publications (30%). Over the past 10 years, manuscripts described implementation outcomes across many settings, emphasizing perceived outcomes like acceptability, feasibility, and appropriateness. We continue to lack robust evidence about strategies that help attain outcomes. Advancing the field demands that the next 10 years further both aims of the 2022 research agenda focusing on building strong theory, more objective measurement, and evidence about how to achieve implementation outcomes. Moreover, we must empirically demonstrate that successful implementation matters for the improvement of clinical service systems and public health outcomes.

### Supplementary Information

**Additional file 1: Supplemental file.**

## Data Availability

Data are available upon reasonable request directed to the corresponding author.
