# Correction: Ten years of implementation outcomes research: a scoping review

**DOI:** 10.1186/s13012-023-01294-z

**Published:** 2023-09-08

**Authors:** Enola K. Proctor, Alicia C. Bunger, Rebecca Lengnick-Hall, Donald R. Gerke, Jared K. Martin, Rebecca J. Phillips, Julia C. Swanson

**Affiliations:** 1 The Brown School, Shanti Khinduka Distinguished Professor Emerita, Washington University in St. Louis, St. Louis, USA; 2 College of Social Work, The Ohio State University, Columbus, OH USA; 3 The Brown School, Washington University in St. Louis, St. Louis, USA; 4 Department of Social Work, College of Arts and Sciences, University of Alabama at Birmingham, Birmingham, USA; 5 College of Education & Human Ecology, The Ohio State University, Columbus, OH USA; 6 College of Liberal Arts & Sciences, Western Oregon University, Monmouth, OR USA; 7 Department of Psychiatry, Washington University School of Medicine, St. Louis, MO USA

**Correction: Implementation Sci 18, 31 (2023)**

**https://doi.org/10.1186/s13012-023-01286-z**

The original publication [1] of this article contained an incorrect citation and a missing reference. The incorrect citation appears in the section entitled “The 2011 call for advances in the conceptualization and measurement”.

In the second paragraph, Lyon and Bruns was incorrectly cited as reference 1, where it should have been cited as reference 70.

The correct reference 70 is supplied below:

70. Lyon AR, Bruns EJ. User-Centered Redesign of Evidence-Based Psychosocial Interventions to Enhance Implementation—Hospitable Soil or Better Seeds? JAMA Psychiatry. 2019;76(1):3–4. doi:10.1001/jamapsychiatry.2018.3060

The original article [1] has been updated.
