# Correction: Ten years of implementation outcomes research: a scoping review

**DOI:** 10.1186/s13012-023-01313-z

**Published:** 2023-10-25

**Authors:** Enola K. Proctor, Alicia C. Bunger, Rebecca Lengnick-Hall, Donald R. Gerke, Jared K. Martin, Rebecca J. Phillips, Julia C. Swanson

**Affiliations:** 1 The Brown School, Shanti Khinduka Distinguished Professor Emerita, Washington University in St. Louis, St. Louis, USA; 2 College of Social Work, The Ohio State University, Columbus, OH USA; 3 The Brown School, Washington University in St. Louis, St. Louis, USA; 4 Department of Social Work, College of Arts and Sciences, University of Alabama at Birmingham, Birmingham, USA; 5 College of Education & Human Ecology, The Ohio State University, Columbus, OH USA; 6 College of Liberal Arts & Sciences, Western Oregon University, Monmouth, OR USA; 7 Department of Psychiatry, Washington University School of Medicine, St. Louis, MO USA

**Correction: Implement Sci 18, 31 (2023)**

**https://doi.org/10.1186/s13012-023-01286-z**

In the original version of this article [1], there was an error in figure labelling. In the section entitled 'Conceptualization of implementation outcomes in the 2011 paper,' it was mentioned that 'the taxonomy is summarized in Fig. 1.' Whereas the Authors intended to convey that the taxonomy was summarized in the said 2011 paper, not in Fig. 1.

Therefore, the correct statement in the article should have read, "…Sustainability is the extent to which an implementation target is maintained or institutionalized within a service setting. The 2011 paper, which originally introduced this taxonomy, encouraged further scholarship of this initial conceptualization, both in terms of the number of outcomes and in further refinements to their operationalization [1]. Cautioning that the original taxonomy included 'only the more obvious,' that paper projected that new concepts would emerge as newly defined implementation outcomes [1].'

The original article [1] has been updated.
