# Supplementary material for: Ten years of implementation outcomes research: a scoping review
Source: Implement Sci. 2023 Jul 25;18:31. doi: 10.1186/s13012-023-01286-z (PMC10367273; doi:10.1186/s13012-023-01286-z)
Supplement: Supplementary file 1 — Additional file 1: Supplemental file. [file 13012_2023_1286_MOESM1_ESM.docx]

1. Aarons GA, Sommerfeld DH. Leadership, Innovation Climate, and Attitudes Toward Evidence-Based Practice During a Statewide Implementation. Journal of the American Academy of Child & Adolescent Psychiatry. 2012;51(4):423-31.

2. Aasmul I, Husebo BS, Flo E. Description of an advance care planning intervention in nursing homes: outcomes of the process evaluation. BMC Geriatr. 2018;18(1):26.

3. Abelson J, Tripp L, Kandasamy S, Burrows K, on behalf of the PIStudy T. Supporting the evaluation of public and patient engagement in health system organizations: Results from an implementation research study. Health Expect. 2019;22(5):1132-43.

4. Abrahamse ME, Niec LN, Junger M, Boer F, Lindauer RJL. Risk factors for attrition from an evidence-based parenting program: Findings from the Netherlands. Children and Youth Services Review. 2016;64:42-50.

5. Affrunti NW, Creed TA. The factor structure of the Cognitive Therapy Rating Scale (CTRS) in a sample of community mental health clinicians. Cognitive Therapy and Research. 2019;43:642-55.

6. Aggarwal NK, Desilva R, Nicasio AV, Boiler M, Lewis-Fernández R. Does the Cultural Formulation Interview for the fifth revision of the diagnostic and statistical manual of mental disorders (DSM-5) affect medical communication? A qualitative exploratory study from the New York site. Ethn Health. 2015;20(1):1-28.

7. Aggarwal NK, Lam P, Diaz S, Cruz AG, Lewis-Fernández R. Clinician Perceptions of Implementing the Cultural Formulation Interview on a Mixed Forensic Unit. J Am Acad Psychiatry Law. 2020;48(2):216-25.

8. Ahuja S, Hanlon C, Chisholm D, Semrau M, Gurung D, Abdulmalik J, et al. Experience of implementing new mental health indicators within information systems in six low- and middle-income countries. BJPsych open. 2019;5(5):e71.

9. Ahuja S, Khan A, Goulding L, Bansal RK, Shidhaye R, Thornicroft G, et al. Evaluation of a new set of indicators for mental health care implemented in Madhya Pradesh, India: a mixed methods study. Int J Ment Health Syst. 2020;14(1):7.

10. Al Achkar M, Bennett IM, Chwastiak L, Hoeft T, Normoyle T, Vredevoogd M, et al. Telepsychiatric Consultation as a Training and Workforce Development Strategy for Rural Primary Care. Ann Fam Med. 2020;18(5):438-45.

11. Allchin B, Weimand BM, O’Hanlon B, Goodyear M. Continued capacity: Factors of importance for organizations to support continued Let's Talk practice – a mixed‐methods study. Int J Mental Health Nurs. 2020;29(6):1131-43.

12. Allicock M, Golin CE, Kaye L, Grodensky C, Blackman LT, Thibodeaux H. SafeTalk: Training Peers to Deliver a Motivational Interviewing HIV Prevention Program. Health Promotion Practice. 2017;18(3):410-7.

13. Allicock M, Haynes-Maslow L, Johnson L-S, Carpenter WR, Vines AI, Belle DG, et al. Peer Connect for African American breast cancer survivors and caregivers: a train-the-trainer approach for peer support. Transl Behav Med. 2017;7(3):495-505.

14. Allison KR, Philipneri AN, Vu-Nguyen K, Manson HE, Dwyer JJM, Hobin E, et al. School and classroom effects on Daily Physical Activity (DPA) policy implementation fidelity in Ontario classrooms: a multi-level analysis. BMC Public Health. 2018;18(1):802.

15. Alvarez GG, Van Dyk DD, Davies N, Aaron SD, Cameron DW, Desjardins M, et al. The Feasibility of the Interferon Gamma Release Assay and Predictors of Discordance with the Tuberculin Skin Test for the Diagnosis of Latent Tuberculosis Infection in a Remote Aboriginal Community. PLoS ONE. 2014;9(11):e111986.

16. Appelhof B, Bakker C, Van Duinen-van Den Ijssel JCL, Zwijsen SA, Smalbrugge M, Verhey FRJ, et al. Process Evaluation of an Intervention for the Management of Neuropsychiatric Symptoms in Young-Onset Dementia. Journal of the American Medical Directors Association. 2018;19(8):663-71.

17. Applegate JA, Ahmed S, Harrison M, Callaghan-Koru J, Mousumi M, Begum N, et al. Caregiver acceptability of the guidelines for managing young infants with possible serious bacterial infections (PSBI) in primary care facilities in rural Bangladesh. PLoS ONE. 2020;15(4):e0231490.

18. Applegate JA, Ahmed S, Harrison M, Callaghan-Koru J, Mousumi M, Begum N, et al. Provider performance and facility readiness for managing infections in young infants in primary care facilities in rural Bangladesh. PLoS ONE. 2020;15(4):e0229988.

19. Arapovic-Johansson B, Jensen I, Wåhlin C, Björklund C, Kwak L. Process Evaluation of a Participative Organizational Intervention as a Stress Preventive Intervention for Employees in Swedish Primary Health Care. IJERPH. 2020;17(19):7285.

20. Ascienzo S, Sprang G, Eslinger J. Disseminating TF‐CBT: A Mixed Methods Investigation of Clinician Perspectives and the Impact of Training Format and Formalized Problem‐Solving Approaches on Implementation Outcomes. J Eval Clin Pract. 2020;26(6):1657-68.

21. Askeland E, Forgatch MS, Apeland A, Reer M, Grønlie AA. Scaling up an Empirically Supported Intervention with Long-Term Outcomes: the Nationwide Implementation of GenerationPMTO in Norway. Prev Sci. 2019;20(8):1189-99.

22. Atkins DC, Steyvers M, Imel ZE, Smyth P. Scaling up the evaluation of psychotherapy: evaluating motivational interviewing fidelity via statistical text classification. Implementation Science. 2014;9(1):49.

23. Auslander W, McGinnis H, Tlapek S, Smith P, Foster A, Edmond T, et al. Adaptation and implementation of a trauma-focused cognitive behavioral intervention for girls in child welfare. American Journal of Orthopsychiatry. 2017;87(3):206-15.

24. Azar KMJ, Nasrallah C, Szwerinski NK, Petersen JJ, Halley MC, Greenwood D, et al. Implementation of a group-based diabetes prevention program within a healthcare delivery system. BMC Health Serv Res. 2019;19(1):694.

25. Bastable E, Meng P, Falcon SF, McIntosh K. Using an Embedded Mixed Methods Design to Assess and Improve Intervention Acceptability of an Equity-Focused Intervention: A Methodological Demonstration. Behavioral Disorders. 2023;48(3):201-11.

26. Baysari MT, Jackson N, Ramasamy S, Santiago P, Xiong J, Westbrook J, et al. Exploring sub-optimal use of an electronic risk assessment tool for venous thromboembolism. Applied Ergonomics. 2016;55:63-9.

27. Bearman SK, Bailin A, Rodriguez E, Bellevue A. Partnering with school providers to codesign mental health interventions: An open trial of Act & Adapt in urban public middle schools. Psychology in the Schools. 2020;57(11):1689-709.

28. Becker-Haimes EM, Williams NJ, Okamura KH, Beidas RS. Interactions Between Clinician and Organizational Characteristics to Predict Cognitive-Behavioral and Psychodynamic Therapy Use. Adm Policy Ment Health. 2019;46(6):701-12.

29. Beidas RS, Edmunds J, Ditty M, Watkins J, Walsh L, Marcus S, et al. Are Inner Context Factors Related to Implementation Outcomes in Cognitive-Behavioral Therapy for Youth Anxiety? Adm Policy Ment Health. 2014;41(6):788-99.

30. Berkowitz CM, Zullig LL, Koontz BF, Smith SK. Prescribing an App? Oncology Providers’ Views on Mobile Health Apps for Cancer Care. JCO Clinical Cancer Informatics. 2017(1):1-7.

31. Berman M, Bozsik F, Shook RP, Meissen-Sebelius E, Markenson D, Summar S, et al. Evaluation of the Healthy Lifestyles Initiative for Improving Community Capacity for Childhood Obesity Prevention. Prev Chronic Dis. 2018;15:170306.

32. Berthold SM, Kong S, Kuoch T, Schilling EA, An R, Blatz M, et al. Combating Health Disparities in Cambodian American Communities: A CBPR Approach to Building Community Capacity. Progress in Community Health Partnerships. 2017;11(2):109-18.

33. Bhattarai AH, Sanjaya GY, Khadka A, Kumar R, Ahmad RA. The addition of mobile SMS effectively improves dengue prevention practices in community: an implementation study in Nepal. BMC Health Serv Res. 2019;19(1):699.

34. Blacklock AS, Meiksans J, Bonser G, Hayden P, Menzies K, Arney F. Acceptability of the Winangay Kinship Carer Assessment Tool. Child Abuse Review. 2018;27(2):108-21.

35. Blaine RE, Franckle RL, Ganter C, Falbe J, Giles C, Criss S, et al. Using School Staff Members to Implement a Childhood Obesity Prevention Intervention in Low-Income School Districts: the Massachusetts Childhood Obesity Research Demonstration (MA-CORD Project), 2012–2014. Prev Chronic Dis. 2017;14:160381.

36. Blake J, Bayliss A, Callow B, Futter G, Harikrishnan N, Peryer G. Using a storytelling intervention in schools to explore death, dying, and loss. JPMH. 2020;19(3):203-11.

37. Blecker S, Austrian JS, Horwitz LI, Kuperman G, Shelley D, Ferrauiola M, et al. Interrupting providers with clinical decision support to improve care for heart failure. International Journal of Medical Informatics. 2019;131:103956.

38. Blok AC, Sadasivam RS, Hogan TP, Patterson A, Day N, Houston TK. Nurse-Driven mHealth Implementation Using the Technology Inpatient Program for Smokers (TIPS): Mixed Methods Study. JMIR Mhealth Uhealth. 2019;7(10):e14331.

39. Boudreau AM, Corkum P, Smith IM. Peer-Mediated Pivotal Response Treatment for Children With Autism Spectrum Disorder: Provider Perspectives on Acceptability, Feasibility, and Fit at School. Canadian Journal of School Psychology. 2019;34(4):259-82.

40. Bozsik F, Berman M, Shook R, Summar S, DeWit E, Carlson J. Implementation contextual factors related to youth advocacy for healthy eating and active living. Transl Behav Med. 2018.

41. Bradley C, Day C, Penney C, Michelson D. ‘Every day is hard, being outside, but you have to do it for your child’: Mixed-methods formative evaluation of a peer-led parenting intervention for homeless families. Clin Child Psychol Psychiatry. 2020;25(4):860-76.

42. Branch-Elliman W, Gupta K, Rani Elwy A. Factors influencing uptake of evidence-based antimicrobial prophylaxis guidelines for electrophysiology procedures. American Journal of Infection Control. 2020;48(6):668-74.

43. Breitenstein SM, Laurent S, Pabalan L, Risser HJ, Roper P, Saba MT, et al. Implementation findings from an effectiveness-implementation trial of tablet-based parent training in pediatric primary care. Families, Systems, & Health. 2019;37(4):282-90.

44. Briere DE, Simonsen B, Sugai G, Myers D. Increasing New Teachers’ Specific Praise Using a Within-School Consultation Intervention. Journal of Positive Behavior Interventions. 2015;17(1):50-60.

45. Brookman-Frazee L, Stadnick N, Roesch S, Regan J, Barnett M, Bando L, et al. Measuring Sustainment of Multiple Practices Fiscally Mandated in Children’s Mental Health Services. Adm Policy Ment Health. 2016;43(6):1009-22.

46. Brookman-Frazee L, Zhan C, Stadnick N, Sommerfeld D, Roesch S, Aarons GA, et al. Using Survival Analysis to Understand Patterns of Sustainment within a System-Driven Implementation of Multiple Evidence-Based Practices for Children’s Mental Health Services. Front Public Health. 2018;6:54.

47. Brown‐Johnson C, Shaw JG, Safaeinili N, Chan GK, Mahoney M, Asch S, et al. Role definition is key— <span style="font-variant:small-caps;">R</span> apid qualitative ethnography findings from a team‐based primary care transformation. Learn Health Sys. 2019;3(3).

48. Brunk MA, Chapman JE, Schoenwald SK. Defining and Evaluating Fidelity at the Program Level in Psychosocial Treatments: A Preliminary Investigation. Zeitschrift für Psychologie. 2014;222(1):22-9.

49. Brunkert T, Simon M, Ruppen W, Zúñiga F. Pain Management in Nursing Home Residents: Findings from a Pilot Effectiveness‐Implementation Study. J Am Geriatr Soc. 2019;67(12):2574-80.

50. Bruns EJ, Parker EM, Hensley S, Pullmann MD, Benjamin PH, Lyon AR, et al. The role of the outer setting in implementation: associations between state demographic, fiscal, and policy factors and use of evidence-based treatments in mental healthcare. Implementation Science. 2019;14(1):96.

51. Burgermaster M, Gray HL, Tipton E, Contento I, Koch P. Testing an Integrated Model of Program Implementation: the Food, Health & Choices School-Based Childhood Obesity Prevention Intervention Process Evaluation. Prev Sci. 2017;18(1):71-82.

52. Bussé AM, Qirjazi B, Goedegebure A, Toll M, Hoeve HL, Toçi E, et al. Implementation of a neonatal hearing screening programme in three provinces in Albania. International Journal of Pediatric Otorhinolaryngology. 2020;134:110039.

53. Byrnes A, Young A, Mudge A, Banks M, Clark D, Bauer J. Prospective application of an implementation framework to improve postoperative nutrition care processes: Evaluation of a mixed methods implementation study: Evaluation of a mixed methods implementation study. Nutr Diet. 2018;75(4):353-62.

54. Caldas SV, Jin L, Dolan M, Dranger P, Contractor AA. An Exploratory Examination of Client Perspectives on a Positive Memory Technique for PTSD. J Nerv Ment Dis. 2020;208(3):230-7.

55. Calo WA, Shah PD, Gilkey MB, Vanderpool RC, Barden S, Doucette WR, et al. Implementing pharmacy-located HPV vaccination: findings from pilot projects in five U.S. states. Human Vaccines & Immunotherapeutics. 2019;15(7-8):1831-8.

56. Carlfjord S, Malmberg E, Skoglund C. Introduction of medication review and medication report in Swedish hospital and primary care, using a theory-based implementation strategy. BMC Health Serv Res. 2020;20(1):867.

57. Carlson JA, Engelberg JK, Cain KL, Conway TL, Geremia C, Bonilla E, et al. Contextual factors related to implementation of classroom physical activity breaks. Transl Behav Med. 2017;7(3):581-92.

58. Carlson JA, Steel C, Bejarano CM, Beauchamp MT, Davis AM, Sallis JF, et al. Walking School Bus Programs: Implementation Factors, Implementation Outcomes, and Student Outcomes, 2017–2018. Prev Chronic Dis. 2020;17:200061.

59. Carron T, Bridevaux P-O, Lörvall K, Parmentier R, Moix J-B, Beytrison V, et al. Feasibility, acceptability and effectiveness of integrated care for COPD patients: a mixed methods evaluation of a pilot community-based programme. Swiss Med Wkly. 2017;147:w14567.

60. Carter JE, Pyati S, Kanach FA, Maxwell AMW, Belden CM, Shea CM, et al. Implementation of Perioperative Music Using the Consolidated Framework for Implementation Research. Anesthesia & Analgesia. 2018;127(3):623-31.

61. Cawthon C, Mion LC, Willens DE, Roumie CL, Kripalani S. Implementing Routine Health Literacy Assessment in Hospital and Primary Care Patients. The Joint Commission Journal on Quality and Patient Safety. 2014;40(2):68-AP1.

62. Chaisson LH, Duong D, Cattamanchi A, Roemer M, Handley MA, Schillinger D, et al. Association of Rapid Molecular Testing With Duration of Respiratory Isolation for Patients With Possible Tuberculosis in a US Hospital. JAMA Intern Med. 2018;178(10):1380.

63. Chambers CT, Dol J, Parker JA, Caes L, Birnie KA, Taddio A, et al. Implementation Effectiveness of a Parent-Directed YouTube Video (“It Doesn’t Have To Hurt”) on Evidence-Based Strategies to Manage Needle Pain: Descriptive Survey Study. JMIR Pediatr Parent. 2020;3(1):e13552.

64. Chard A, Freeman M. Design, Intervention Fidelity, and Behavioral Outcomes of a School-Based Water, Sanitation, and Hygiene Cluster-Randomized Trial in Laos. IJERPH. 2018;15(4):570.

65. Chard AN, Garn JV, Chang HH, Clasen T, Freeman MC. Impact of a school-based water, sanitation, and hygiene intervention on school absence, diarrhea, respiratory infection, and soil-transmitted helminths: results from the WASH HELPS cluster-randomized trial. J Glob Health.9(2):020402.

66. Charest É, Gagné M-H. Measuring and Predicting Service Providers’ Use of an Evidence-Based Parenting Program. Adm Policy Ment Health. 2019;46(4):542-54.

67. Cheek C, Bridgman H, Fleming T, Cummings E, Ellis L, Lucassen MF, et al. Views of Young People in Rural Australia on SPARX, a Fantasy World Developed for New Zealand Youth With Depression. JMIR Serious Games. 2014;2(1):e3.

68. Chen S, Kraak VI, Price TT, Love K, Byker Shanks C, Serrano EL. Exploring Food Waste at a Residential Youth Summer Camp: A Mixed-Methods Study. Journal of Hunger & Environmental Nutrition. 2022;17(1):135-48.

69. Chesnut RP, Czymoniewicz-Klippel M, DiNallo JM, Perkins DF. Grow Online: feasibility and proof of concept study. Journal of Children's Services. 2019;15(1):25-42.

70. Clack L, Zingg W, Saint S, Casillas A, Touveneau S, Da Liberdade Jantarada F, et al. Implementing infection prevention practices across European hospitals: an in-depth qualitative assessment. BMJ Qual Saf. 2018;27(10):771-80.

71. Cook CR, Xie SR, Earl RK, Lyon AR, Dart E, Zhang Y. Evaluation of the Courage and Confidence Mentor Program as a Tier 2 Intervention for Middle School Students with Identified Internalizing Problems. School Mental Health. 2015;7(2):132-46.

72. Cook JM, Dinnen S, Thompson R, Ruzek J, Coyne JC, Schnurr PP. A Quantitative Test of an Implementation Framework in 38 VA Residential PTSD Programs. Adm Policy Ment Health. 2015;42(4):462-73.

73. Couture M, Israël S, Soulières M, Sasseville M. Implementing a Systematic Screening Procedure for Older Adult Mistreatment Within Individual Clinical Supervision: Is It Feasible? J Interpers Violence. 2019;34(13):2813-33.

74. Coventry P, Bower P, Blakemore A, Baker E, Hann M, Li J, et al. Satisfaction with a digitally-enabled telephone health coaching intervention for people with non-diabetic hyperglycaemia. npj Digital Med. 2019;2(1):5.

75. Creed TA, Frankel SA, German RE, Green KL, Jager-Hyman S, Taylor KP, et al. Implementation of transdiagnostic cognitive therapy in community behavioral health: The Beck Community Initiative. Journal of Consulting and Clinical Psychology. 2016;84(12):1116-26.

76. Cross W, West J, Wyman PA, Schmeelk-Cone K, Xia Y, Tu X, et al. Observational Measures of Implementer Fidelity for a School-Based Preventive Intervention: Development, Reliability, and Validity. Prev Sci. 2015;16(1):122-32.

77. Czymoniewicz-Klippel M, Chesnut R, DiNallo J, Perkins D. Patterns of participation in the Grow parenting program. Journal of Children's Services. 2019;14(1):27-41.

78. Czymoniewicz-Klippel MT, Chesnut RP, DiNallo JM, Perkins DF. Understanding the implementation of the parenting program: Findings from a mixed methods pilot study. Children and Youth Services Review. 2017;82:99-107.

79. Damschroder LJ, Goodrich DE, Kim HM, Holleman R, Gillon L, Kirsh S, et al. Development and validation of the ASPIRE-VA coaching fidelity checklist (ACFC): a tool to help ensure delivery of high-quality weight management interventions. Transl Behav Med. 2016;6(3):369-85.

80. Danitz SB, Stirman SW, Grillo AR, Dichter ME, Driscoll M, Gerber MR, et al. When user-centered design meets implementation science: integrating provider perspectives in the development of an intimate partner violence intervention for women treated in the United States’ largest integrated healthcare system. BMC Women's Health. 2019;19(1):145.

81. Dauber S, John T, Hogue A, Nugent J, Hernandez G. Development and implementation of a screen-and-refer approach to addressing maternal depression, substance use, and intimate partner violence in home visiting clients. Children and Youth Services Review. 2017;81:157-67.

82. De Beuf TLF, De Vogel V, De Ruiter C. Adherence to Structured Risk Assessment Guidelines: Development and Preliminary Evaluation of an Adherence Scale for the START:AV. Journal of Forensic Psychology Research and Practice. 2020;20(5):413-35.

83. De Marchis EH, Hessler D, Fichtenberg C, Adler N, Byhoff E, Cohen AJ, et al. Part I: A Quantitative Study of Social Risk Screening Acceptability in Patients and Caregivers. American Journal of Preventive Medicine. 2019;57(6):S25-S37.

84. Dickson-Gomez J, Tarima S, Glasman LR, Lechuga J, Bodnar G, De Mendoza LR. Intervention Reach and Sexual Risk Reduction of a Multi-level, Community-Based HIV Prevention Intervention for Crack Users in San Salvador, El Salvador. AIDS Behav. 2019;23(5):1147-57.

85. Diebold A, Segovia M, Johnson JK, Degillio A, Zakieh D, Park HJ, et al. Acceptability and appropriateness of a perinatal depression preventive group intervention: a qualitative analysis. BMC Health Serv Res. 2020;20(1):189.

86. Diez-Canseco F, Rojas-Vargas J, Toyama M, Mendoza M, Cavero V, Maldonado H, et al. Qualitative study of the implementation of the Continuity of Care and Rehabilitation Program for people with severe mental disorders in Peru. Revista Panamericana de Salud Pública. 2020;44:1.

87. Diffin J, Ewing G, Harvey G, Grande G. The Influence of Context and Practitioner Attitudes on Implementation of Person‐Centered Assessment and Support for Family Carers Within Palliative Care. Worldviews on Evidence‐Based Nursing. 2018;15(5):377-85.

88. Doherty E, Wiggers J, Wolfenden L, Anderson AE, Crooks K, Tsang TW, et al. Antenatal care for alcohol consumption during pregnancy: pregnant women’s reported receipt of care and associated characteristics. BMC Pregnancy and Childbirth. 2019;19(1):299.

89. Domitrovich CE, Li Y, Mathis ET, Greenberg MT. Individual and organizational factors associated with teacher self-reported implementation of the PATHS curriculum. Journal of School Psychology. 2019;76:168-85.

90. Donenberg GR, Cohen MH, Ingabire C, Fabri M, Emerson E, Kendall AD, et al. Applying the Exploration Preparation Implementation Sustainment (EPIS) Framework to the Kigali Imbereheza Project for Rwandan Adolescents Living With HIV. JAIDS Journal of Acquired Immune Deficiency Syndromes. 2019;82(3):S289-S98.

91. Donnelly KZ, Baker K, Pierce R, St. Ivany AR, Barr PJ, Bruce ML. A retrospective study on the acceptability, feasibility, and effectiveness of LoveYourBrain Yoga for people with traumatic brain injury and caregivers. Disability and Rehabilitation. 2021;43(12):1764-75.

92. Dopp AR, Coen AS, Smith AB, Reno J, Bernstein DH, Kerns SEU, et al. Economic Impact of the Statewide Implementation of an Evidence-Based Treatment: Multisystemic Therapy in New Mexico. Behavior Therapy. 2018;49(4):551-66.

93. Dopp AR, Mundey P, Silovsky JF, Hunter M, Slemaker A. Economic value of community-based services for problematic sexual behaviors in youth: A mixed-method cost-effectiveness analysis. Child Abuse & Neglect. 2020;105:104043.

94. Dorsey S, Lyon AR, Pullmann MD, Jungbluth N, Berliner L, Beidas R. Behavioral Rehearsal for Analogue Fidelity: Feasibility in a State-Funded Children’s Mental Health Initiative. Adm Policy Ment Health. 2017;44(3):395-404.

95. Dorsey S, Meza RD, Martin P, Gray CL, Triplett NS, Soi C, et al. Lay Counselor Perspectives of Providing a Child-Focused Mental Health Intervention for Children: Task-Shifting in the Education and Health Sectors in Kenya. Front Psychiatry. 2019;10:860.

96. Drmic IE, Aljunied M, Reaven J. Feasibility, Acceptability and Preliminary Treatment Outcomes in a School-Based CBT Intervention Program for Adolescents with ASD and Anxiety in Singapore. J Autism Dev Disord. 2017;47(12):3909-29.

97. Dugan AG, Farr DA, Namazi S, Henning RA, Wallace KN, El Ghaziri M, et al. Process evaluation of two participatory approaches: Implementing total worker health® interventions in a correctional workforce: Two Participatory Programs in Corrections. Am J Ind Med. 2016;59(10):897-918.

98. Dulude E, Coutu M-F, Durand M-J. Promoting resilience in work rehabilitation: development of a transdiagnostic intervention. Disability and Rehabilitation. 2021;43(25):3652-62.

99. Duong MT, Lyon AR, Ludwig K, Wasse JK, McCauley E. Student perceptions of the acceptability and utility of standardized and idiographic assessment in school mental health. International Journal of Mental Health Promotion. 2016;18(1):49-63.

100. Dutta O, Lall P, Patinadan PV, Car J, Low CK, Tan WS, et al. Patient autonomy and participation in end-of-life decision-making: An interpretive-systemic focus group study on perspectives of Asian healthcare professionals. Pall Supp Care. 2020;18(4):425-30.

101. Edmunds JM, Brodman DM, Ringle VA, Read KL, Kendall PC, Beidas RS. Examining adherence to components of cognitive-behavioral therapy for youth anxiety after training and consultation. Professional Psychology: Research and Practice. 2017;48(1):54-61.

102. Edmunds JM, Read KL, Ringle VA, Brodman DM, Kendall PC, Beidas RS. Sustaining clinician penetration, attitudes and knowledge in cognitive-behavioral therapy for youth anxiety. Implementation Science. 2014;9(1):89.

103. Egeland KM, Heiervang KS, Landers M, Ruud T, Drake RE, Bond GR. Psychometric Properties of a Fidelity Scale for Illness Management and Recovery. Adm Policy Ment Health. 2020;47(6):885-93.

104. Egeland KM, Ruud T, Ogden T, Färdig R, Lindstrøm JC, Heiervang KS. How to implement Illness Management and Recovery (IMR) in mental health service settings: evaluation of the implementation strategy. Int J Ment Health Syst. 2017;11(1):13.

105. Eisman AB, Kilbourne AM, Greene D, Walton M, Cunningham R. The User-Program Interaction: How Teacher Experience Shapes the Relationship Between Intervention Packaging and Fidelity to a State-Adopted Health Curriculum. Prev Sci. 2020;21(6):820-9.

106. Eisman AB, Kilbourne AM, Ngo Q, Fridline J, Zimmerman MA, Greene D, et al. Implementing a State‐Adopted High School Health Curriculum: A Case Study. J School Health. 2020;90(6):447-56.

107. Elbers S, Pool J, Wittink H, Köke A, Smeets R. Exploring the Feasibility of Relapse Prevention Strategies in Interdisciplinary Multimodal Pain Therapy Programs: Qualitative Study. JMIR Hum Factors. 2020;7(4):e21545.

108. Elkington KS, Spaulding A, Gardner S, Knight D, Belenko S, Becan JE, et al. A System-Level Intervention to Encourage Collaboration Between Juvenile Justice and Public Health Agencies to Promote HIV/STI Testing. AIDS Education and Prevention. 2020;32(4):337-55.

109. Ervin JN. An acceptability pilot of the facilitating active management in lung illness with engaged surrogates (FAMILIES) study. Medicine. 2020;99(9):e19272.

110. Fahim C, Acai A, McConnell MM, Wright FC, Sonnadara RR, Simunovic M. Use of the theoretical domains framework and behaviour change wheel to develop a novel intervention to improve the quality of multidisciplinary cancer conference decision-making. BMC Health Serv Res. 2020;20(1):578.

111. Fenwick K, Henwood B, Lengnick-Hall R, Stefancic A, Gilmer T. Exploring Variation in Housing First Implementation: The Role of Fit. Human Service Organizations: Management, Leadership & Governance. 2019;43(5):392-406.

112. Feuerstein J, Olswang LB, Greenslade K, Pinder GL, Dowden P, Madden J. Moving Triadic Gaze Intervention Into Practice: Measuring Clinician Attitude and Implementation Fidelity. J Speech Lang Hear Res. 2017;60(5):1285-98.

113. Feuerstein JL, Olswang LB, Greenslade KJ, Dowden P, Pinder GL, Madden J. Implementation Research: Embracing Practitioners' Views. J Speech Lang Hear Res. 2018;61(3):645-57.

114. Fiat AE, Cook CR, Zhang Y, Renshaw TL, DeCano P, Merrick JS. Mentoring to Promote Courage and Confidence Among Elementary School Students With Internalizing Problems: A Single-Case Design Pilot Study. Journal of Applied School Psychology. 2017;33(4):261-87.

115. Flores EJ, Mull NK, Lavenberg JG, Mitchell MD, Leas BF, Williams A, et al. Using a 10-step framework to support the implementation of an evidence-based clinical pathways programme. BMJ Qual Saf. 2019;28(6):476-85.

116. Folk JB, Blasko BL, Warden R, Schaefer K, Ferssizidis P, Stuewig J, et al. Feasibility and Acceptability of an Impact of Crime Group Intervention with Jail Inmates. Victims & Offenders. 2016;11(3):436-54.

117. Frey AJ, Kuklinski MR, Bills K, Small JW, Forness SR, Walker HM, et al. Comprehensive Cost Analysis of First Step Next for Preschoolers with Disruptive Behavior Disorder: Using Real-World Intervention Data to Estimate Costs at Scale. Prev Sci. 2019;20(8):1219-32.

118. Fuchs CH, Haradhvala N, Evans DR, Nash JM, Weisberg RB, Uebelacker LA. Implementation of an acceptance- and mindfulness-based group for depression and anxiety in primary care: Initial outcomes. Families, Systems, & Health. 2016;34(4):386-95.

119. Fulop NJ, Ramsay AIG, Perry C, Boaden RJ, McKevitt C, Rudd AG, et al. Explaining outcomes in major system change: a qualitative study of implementing centralised acute stroke services in two large metropolitan regions in England. Implementation Science. 2015;11(1):80.

120. Gaitán-Rossi P, De La Cerda Lobato S, Pérez Navarro AC, Aguilar Esteva A, Vargas García MR, Vilar-Compte M. Fidelity of Implementation of Prospera Digital: Evaluation of a Multi-Site mHealth Intervention Aimed at Improving Maternal Health Outcomes in Mexico. Current Developments in Nutrition. 2019;3(10):nzz107.

121. Garcia-Cardenas V, Benrimoj SI, Ocampo CC, Goyenechea E, Martinez–Martinez F, Gastelurrutia MA. Evaluation of the implementation process and outcomes of a professional pharmacy service in a community pharmacy setting. A case report. Research in Social and Administrative Pharmacy. 2017;13(3):614-27.

122. Garner BR, Hunter SB, Funk RR, Griffin BA, Godley SH. Toward Evidence-Based Measures of Implementation: Examining the Relationship Between Implementation Outcomes and Client Outcomes. Journal of Substance Abuse Treatment. 2016;67:15-21.

123. Garner BR, Hunter SB, Slaughter ME, Han B, Godley SH. Factors associated with an evidence-based measure of implementation for the Adolescent Community Reinforcement Approach. Drug and Alcohol Dependence. 2017;180:144-50.

124. Geerligs L, Shepherd HL, Rankin NM, Masya L, Shaw JM, Price MA, et al. The value of real-world testing: a qualitative feasibility study to explore staff and organisational barriers and strategies to support implementation of a clinical pathway for the management of anxiety and depression in adult cancer patients. Pilot Feasibility Stud. 2020;6(1):109.

125. Gélinas C, Ross M, Boitor M, Desjardins S, Vaillant F, Michaud C. Nurses' evaluations of the CPOT use at 12-month post-implementation in the intensive care unit: ICU nurses' evaluations of the CPOT use. Nurs Crit Care. 2014;19(6):272-80.

126. Gichane MW, Wechsberg WM, Ndirangu J, Browne FA, Bonner CP, Grimwood A, et al. Implementation science outcomes of a gender-focused HIV and alcohol risk-reduction intervention in usual-care settings in South Africa. Drug and Alcohol Dependence. 2020;215:108206.

127. Gold R, Bunce A, Cowburn S, Davis JV, Nelson JC, Nelson CA, et al. Does increased implementation support improve community clinics’ guideline-concordant care? Results of a mixed methods, pragmatic comparative effectiveness trial. Implementation Science. 2019;14(1):100.

128. Gonzalez-Viana A, Violan Fors M, Castell Abat C, Rubinat Masot M, Oliveras L, Garcia-Gil J, et al. Promoting physical activity through primary health care: the case of Catalonia. BMC Public Health. 2018;18(1):968.

129. Guidi JL, Clark K, Upton MT, Faust H, Umscheid CA, Lane-Fall MB, et al. Clinician Perception of the Effectiveness of an Automated Early Warning and Response System for Sepsis in an Academic Medical Center. Annals ATS. 2015;12(10):1514-9.

130. Guy LS, Nelson RJ, Fusco-Morin SL, Vincent GM. What Do Juvenile Probation Officers Think of Using the SAVRY and YLS/CMI for Case Management, and Do They Use the Instruments Properly? International Journal of Forensic Mental Health. 2014;13(3):227-41.

131. Hackett K, Huber-Krum S, Francis JM, Senderowicz L, Pearson E, Siril H, et al. Evaluating the Implementation of an Intervention to Improve Postpartum Contraception in Tanzania: A Qualitative Study of Provider and Client Perspectives. Global Health: Science and Practice. 2020;8(2):270-89.

132. Haine-Schlagel R, Brookman-Frazee L, Janis B, Gordon J. Evaluating a Learning Collaborative to Implement Evidence-Informed Engagement Strategies in Community-Based Services for Young Children. Child Youth Care Forum. 2013;42(5):457-73.

133. Haine-Schlagel R, Fettes DL, Finn N, Hurlburt M, Aarons GA. Parent and Caregiver Active Participation Toolkit (PACT): Adaptation for a Home Visitation Program. Journal of Child and Family Studies. 2020;29(1):29-43.

134. Haine-Schlagel R, Mechammil M, Brookman-Frazee L. Stakeholder perspectives on a toolkit to enhance caregiver participation in community-based child mental health services. Psychological Services. 2017;14(3):373-86.

135. Haine‐Schlagel R, Rieth S, Dickson KS, Brookman‐Frazee L, Stahmer A. Adapting parent engagement strategies for an evidence‐based parent‐mediated intervention for young children at risk for autism spectrum disorder. Journal of Community Psychology. 2020;48(4):1215-37.

136. Hammarström S, Nilsen P, Lindroth M, Stenqvist K, Bernhardsson S. Identifying young people exposed to or at risk of sexual ill health: pilot implementation of an evidence-informed toolkit (SEXIT) at Swedish youth clinics. The European Journal of Contraception & Reproductive Health Care. 2019;24(1):45-53.

137. Hartzler B, Dombrowski JC, Donovan DM. Contextual compatibility of three empirically supported behavior therapies for cART adherence among patients with substance use disorders. AIDS Care. 2019;31(1):19-24.

138. Hartzler B, Jackson TR, Jones BE, Beadnell B, Calsyn DA. Disseminating contingency management: Impacts of staff training and implementation at an opiate treatment program. Journal of Substance Abuse Treatment. 2014;46(4):429-38.

139. Hearld LR, Carroll N, Hall A. The adoption and spread of hospital care coordination activities under value-based programs. Am J Manag Care. 2019;25(8):397-404.

140. Helseth SA, Peer SO, Are F, Korell AM, Saunders BE, Schoenwald SK, et al. Sustainment of Trauma-Focused and Evidence-Based Practices Following Learning Collaborative Implementation. Adm Policy Ment Health. 2020;47(4):569-80.

141. Henriksen L, Ribisl KM, Rogers T, Moreland-Russell S, Barker DM, Sarris Esquivel N, et al. Standardized Tobacco Assessment for Retail Settings (STARS): dissemination and implementation research. Tob Control. 2016;25(Suppl 1):i67-i74.

142. Herr K, Titler M, Fine PG, Sanders S, Cavanaugh JE, Swegle J, et al. The Effect of a Translating Research into Practice (TRIP)-Cancer Intervention on Cancer Pain Management in Older Adults in Hospice. Pain Med. 2012;13(8):1004-17.

143. Herschell AD, Quetsch LB, Kolko DJ. Measuring Adherence to Key Teaching Techniques in an Evidence-Based Treatment: A Comparison of Caregiver, Therapist, and Behavior Observation Ratings. Journal of Emotional and Behavioral Disorders. 2020;28(2):92-103.

144. Hickey G, McGilloway S, Leckey Y, Stokes A, Bywater T, Donnelly M. “Putting Meat on the Bones”: Understanding the Implementation of a Community-Based Early Intervention and Prevention Programme—Contextual, Person, and Programme Influences. Prev Sci. 2021;22(1):113-29.

145. Hirsch O, Keller H, Krones T, Donner-Banzhoff N. arriba-lib: evaluation of an electronic library of decision aids in primary care physicians. BMC Med Inform Decis Mak. 2012;12(1):48.

146. Hoben M, Estabrooks CA, Squires JE, Behrens J. Factor Structure, Reliability and Measurement Invariance of the Alberta Context Tool and the Conceptual Research Utilization Scale, for German Residential Long Term Care. Front Psychol. 2016;7.

147. Holdsworth LM, Zionts D, Wang S, Veruttipong D, Brown-Johnson C, Asch SM, et al. Negotiating Lay and Clinical Issues: Implementing a Lay Navigation Program in Cancer Care. JCO Oncology Practice. 2020;16(1):e84-e91.

148. Holtrop K, Canto AI, Schelbe L, McWey LM, Radey M, Montgomery JE. Adapting a parenting intervention for parents aging out of the child welfare system: A systematic approach to expand the reach of an evidence-based intervention. American Journal of Orthopsychiatry. 2018;88(3):386-98.

149. Holtrop K, Holcomb JE. Adapting and Pilot Testing a Parenting Intervention for Homeless Families in Transitional Housing. Fam Proc. 2018;57(4):884-900.

150. Houghtaling B, Serrano E, Dobson L, Chen S, Kraak VI, Harden SM, et al. Rural independent and corporate Supplemental Nutrition Assistance Program (SNAP)-authorized store owners’ and managers’ perceived feasibility to implement marketing-mix and choice-architecture strategies to encourage healthy consumer purchases. Transl Behav Med. 2019;9(5):888-98.

151. Howell D, Rosberger Z, Mayer C, Faria R, Hamel M, Snider A, et al. Personalized symptom management: a quality improvement collaborative for implementation of patient reported outcomes (PROs) in ‘real-world’ oncology multisite practices. J Patient Rep Outcomes. 2020;4(1):47.

152. Hunter SB, Han B, Slaughter ME, Godley SH, Garner BR. Predicting evidence-based treatment sustainment: results from a longitudinal study of the Adolescent-Community Reinforcement Approach. Implementation Science. 2017;12(1):75.

153. Hunter SB, Ober AJ, McCullough CM, Storholm ED, Iyiewuare PO, Pham C, et al. Sustaining alcohol and opioid use disorder treatment in primary care: a mixed methods study. Implementation Science. 2018;13(1):83.

154. Hunter SB, Rutter CM, Ober AJ, Booth MS. Building capacity for continuous quality improvement (CQI): A pilot study. Journal of Substance Abuse Treatment. 2017;81:44-52.

155. Ihorn SM, Arora P. Teleconsultation to Support the Education of Students with Visual Impairments: A Program Evaluation. Journal of Educational and Psychological Consultation. 2018;28(3):319-41.

156. Ireys HT, Brach C, Anglin G, Devers KJ, Burton R. After The Demonstration: What States Sustained After the End of Federal Grants to Improve Children’s Health Care Quality. Matern Child Health J. 2018;22(2):195-203.

157. Irving A, Buykx P, Amos Y, Goodacre S, Moore SC, O'Cathain A. The acceptability of alcohol intoxication management services to users: A mixed methods study. Drug Alcohol Rev. 2020;39(1):36-43.

158. Iverson KM, Sorrentino AE, Bellamy SL, Grillo AR, Haywood TN, Medvedeva E, et al. Adoption, penetration, and effectiveness of a secondary risk screener for intimate partner violence: Evidence to inform screening practices in integrated care settings. General Hospital Psychiatry. 2018;51:79-84.

159. Jack HE, Merritt C, Medhin G, Musesengwa R, Mafuta C, Gibson LJ, et al. Developing sustainable capacity-building in mental health research: implementation outcomes of training of trainers in systematic reviewing. Global Health Action. 2020;13(1):1715325.

160. Jackson CB, Herschell AD, Schaffner KF, Turiano NA, McNeil CB. Training community-based clinicians in parent-child interaction therapy: The interaction between expert consultation and caseload. Professional Psychology: Research and Practice. 2017;48(6):481-9.

161. Jacobs RH, Guo S, Kaundinya P, Lakind D, Klein J, Rusch D, et al. A Pilot Study of Mindfulness Skills to Reduce Stress among a Diverse Paraprofessional Workforce. Journal of Child and Family Studies. 2017;26(9):2579-88.

162. Jacobson N, Horst J, Wilcox-Warren L, Toy A, Knudsen HK, Brown R, et al. Organizational Facilitators and Barriers to Medication for Opioid Use Disorder Capacity Expansion and Use. J Behav Health Serv Res. 2020;47(4):439-48.

163. James S, Thompson RW, Ringle JL. The Implementation of Evidence-Based Practices in Residential Care: Outcomes, Processes, and Barriers. Journal of Emotional and Behavioral Disorders. 2017;25(1):4-18.

164. Jarrett BA, Woznica DM, Tilchin C, Mpungose N, Motlhaoleng K, Golub JE, et al. Promoting Tuberculosis Preventive Therapy for People Living with HIV in South Africa: Interventions Hindered by Complicated Clinical Guidelines and Imbalanced Patient-Provider Dynamics. AIDS Behav. 2020;24(4):1106-17.

165. Jayes M, Palmer R, Enderby P. Evaluation of the MCAST, a multidisciplinary toolkit to improve mental capacity assessment. Disability and Rehabilitation. 2022;44(2):323-30.

166. Johanson S, Markström U, Larsson ME, Bejerholm U. Implementation of a novel return-to-work approach for persons with affective disorders in a traditional vocational rehabilitation context: a case study. Int J Ment Health Syst. 2020;14(1):22.

167. Johnson JE, Hailemariam M, Zlotnick C, Richie F, Sinclair J, Chuong A, et al. Mixed Methods Analysis of Implementation of Interpersonal Psychotherapy (IPT) for Major Depressive Disorder in Prisons in a Hybrid Type I Randomized Trial. Adm Policy Ment Health. 2020;47(3):410-26.

168. Johnson K, Collins D, Shamblen S, Kenworthy T, Wandersman A. Long-Term Sustainability of Evidence-Based Prevention Interventions and Community Coalitions Survival: a Five and One-Half Year Follow-up Study. Prev Sci. 2017;18(5):610-21.

169. Joseph C, Leavy B, Mattsson S, Falk L, Franzén E. Implementation of the HiBalance training program for Parkinson’s disease in clinical settings: A feasibility study. Brain Behav. 2018;8(8):e01021.

170. Kanuri N, Arora P, Talluru S, Colaco B, Dutta R, Rawat A, et al. Examining the initial usability, acceptability and feasibility of a digital mental health intervention for college students in India. Int J Psychol. 2020;55(4):657-73.

171. Keen D, Paynter J, Simpson K, Sulek R, Trembath D. Implementing structured consultation with autism spectrum disorder early intervention practitioners. Journal of Intellectual & Developmental Disability. 2017;42(3):269-74.

172. Kerns SEU, McCormick E, Negrete A, Carey C, Haaland W, Waller S. Predicting post-training implementation of a parenting intervention. Journal of Children's Services. 2017;12(4):302-15.

173. Khenti A, Thomas FC, Mohamoud S, Diaz P, Vaccarino O, Dunbar K, et al. Mental health and addictions capacity building for community health centres in Ontario. Can Fam Physician. 2017;63(10):e416-e24.

174. Kia‐Keating M, Barnett ML, Liu SR, Sims GM, Ruth AB. Trauma‐Responsive Care in a Pediatric Setting: Feasibility and Acceptability of Screening for Adverse Childhood Experiences. Am J Community Psychol. 2019;64(3-4):286-97.

175. Kibel M, Shah P, Ayuku D, Makori D, Kamaara E, Choge E, et al. Acceptability of a Pilot Intervention of Voluntary Medical Male Circumcision and HIV Education for Street-Connected Youth in Western Kenya. Journal of Adolescent Health. 2019;64(1):43-8.

176. Kilburn JE, Shapiro CJ, Hardin JW. Linking implementation of evidence-based parenting programs to outcomes in early intervention. Research in Developmental Disabilities. 2017;70:50-8.

177. King DK, Shoup JA, Raebel MA, Anderson CB, Wagner NM, Ritzwoller DP, et al. Planning for Implementation Success Using RE-AIM and CFIR Frameworks: A Qualitative Study. Front Public Health. 2020;8:59.

178. King ES, Moore CJ, Wilson HK, Harden SM, Davis M, Berg AC. Mixed methods evaluation of implementation and outcomes in a community-based cancer prevention intervention. BMC Public Health. 2019;19(1):1051.

179. King JC, Hibbs R, Saville CWN, Swales MA. The survivability of dialectical behaviour therapy programmes: a mixed methods analysis of barriers and facilitators to implementation within UK healthcare settings. BMC Psychiatry. 2018;18(1):302.

180. Kingma AEC, Van Stel HF, Oudega R, Moons KGM, Geersing G-J. Multi-faceted implementation strategy to increase use of a clinical guideline for the diagnosis of deep venous thrombosis in primary care. FAMPRJ. 2016:cmw066.

181. Kip H, Sieverink F, Van Gemert-Pijnen LJEWC, Bouman YHA, Kelders SM. Integrating People, Context, and Technology in the Implementation of a Web-Based Intervention in Forensic Mental Health Care: Mixed-Methods Study. J Med Internet Res. 2020;22(5):e16906.

182. Kittelman A, McIntosh K, Hoselton R. Adoption of PBIS within school districts. Journal of School Psychology. 2019;76:159-67.

183. Kleiber BV, Felder JN, Ashby B, Scott S, Dean J, Dimidjian S. Treating Depression Among Adolescent Perinatal Women With a Dialectical Behavior Therapy–Informed Skills Group. Cognitive and Behavioral Practice. 2017;24(4):416-27.

184. Klemm GC, Birhanu Z, Ortolano SE, Kebede Y, Martin SL, Mamo G, et al. Integrating Calcium Into Antenatal Iron-Folic Acid Supplementation in Ethiopia: Women’s Experiences, Perceptions of Acceptability, and Strategies to Support Calcium Supplement Adherence. Global Health: Science and Practice. 2020;8(3):413-30.

185. Kolko RP, Kass AE, Hayes JF, Levine MD, Garbutt JM, Proctor EK, et al. Provider Training to Screen and Initiate Evidence-Based Pediatric Obesity Treatment in Routine Practice Settings: A Randomized Pilot Trial. Journal of Pediatric Health Care. 2017;31(1):16-28.

186. Kopelovich SL, Hughes M, Monroe-DeVita MB, Peterson R, Cather C, Gottlieb J. Statewide Implementation of Cognitive Behavioral Therapy for Psychosis Through a Learning Collaborative Model. Cognitive and Behavioral Practice. 2019;26(3):439-52.

187. Korchmaros JD, Greene A, Murphy S. Implementing Trauma-Informed Research-Supported Treatment: Fidelity, Feasibility, and Acceptability. Child Adolesc Soc Work J. 2021;38(1):101-13.

188. Krist AH, Phillips SM, Sabo RT, Balasubramanian BA, Heurtin-Roberts S, Ory MG, et al. Adoption, Reach, Implementation, and Maintenance of a Behavioral and Mental Health Assessment in Primary Care. Ann Fam Med. 2014;12(6):525-33.

189. Kubiak S, Shamrova D, Comartin E. Enhancing knowledge of adolescent mental health among law enforcement: Implementing youth-focused crisis intervention team training. Evaluation and Program Planning. 2019;73:44-52.

190. Kumar R, Probandari A, Ojha B, Bhattarai AH, Subronto YW. Implementation fidelity of provider-initiated HIV testing and counseling of tuberculosis patients under the National Tuberculosis Control Program in Kathmandu District of Nepal: an implementation research. BMC Health Serv Res. 2019;19(1):543.

191. Kwak L, Wåhlin C, Stigmar K, Jensen I. Developing a practice guideline for the occupational health services by using a community of practice approach: a process evaluation of the development process. BMC Public Health. 2017;17(1):89.

192. Lamontagne M-E, Gargaro J, Marier-Deschênes P, Truchon C, Bayley MT, Marshall S, et al. A Survey of Perceived Implementation Gaps for a Clinical Practice Guideline for the Rehabilitation of Adults With Moderate to Severe Traumatic Brain Injury. Journal of Head Trauma Rehabilitation. 2018;33(5):306-16.

193. Lang JM, Connell CM. Measuring Costs to Community-Based Agencies for Implementation of an Evidence-Based Practice. J Behav Health Serv Res. 2017;44(1):122-34.

194. Lau AS, Lind T, Crawley M, Rodriguez A, Smith A, Brookman-Frazee L. When Do Therapists Stop Using Evidence-Based Practices? Findings from a Mixed Method Study on System-Driven Implementation of Multiple EBPs for Children. Adm Policy Ment Health. 2020;47(2):323-37.

195. Leadbeater B, Thompson K, Sukhawathanakul P, Merrin GJ. How Program Users Enhance Fidelity: Implementing the WITS Programs in Rural Canadian Elementary Schools. Prevention Science: The Official Journal of the Society for Prevention Research. 2018;19(8):1066-78.

196. Lebina L, Alaba O, Ringane A, Hlongwane K, Pule P, Oni T, et al. Process evaluation of implementation fidelity of the integrated chronic disease management model in two districts, South Africa. BMC Health Serv Res. 2019;19(1):965.

197. Leeman J, Petermann V, Heisler-MacKinnon J, Bjork A, Brewer NT, Grabert BK, et al. Quality Improvement Coaching for Human Papillomavirus Vaccination Coverage: A Process Evaluation in 3 States, 2018–2019. Prev Chronic Dis. 2020;17:190410.

198. Leppin AL, Boehmer KR, Branda ME, Shah ND, Hargraves I, Dick S, et al. Developing a toolkit to implement the Statin Choice Conversation Aid at scale: application of a work reduction model. BMC Health Serv Res. 2019;19(1):249.

199. Levy NK, Orzeck-Byrnes NA, Aidasani SR, Moloney DN, Nguyen LH, Park A, et al. Transition of a Text-Based Insulin Titration Program From a Randomized Controlled Trial Into Real-World Settings: Implementation Study. J Med Internet Res. 2018;20(3):e93.

200. Lewis EM, Feely M, Seay KD, Fedoravicis N, Kohl PL. Child Welfare Involved Parents and Pathways Triple P:Perceptions of Program Acceptability and Appropriateness. Journal of Child and Family Studies. 2016;25(12):3760-70.

201. Lewis-Fernández R, Aggarwal NK, Lam PC, Galfalvy H, Weiss MG, Kirmayer LJ, et al. Feasibility, acceptability and clinical utility of the Cultural Formulation Interview: Mixed-methods results from the DSM-5 international field trial. Br J Psychiatry. 2017;210(4):290-7.

202. Lindgren S, Wacker D, Suess A, Schieltz K, Pelzel K, Kopelman T, et al. Telehealth and Autism: Treating Challenging Behavior at Lower Cost. Pediatrics. 2016;137(Supplement_2):S167-S75.

203. Lindholm LH, Koivukangas A, Lassila A, Kampman O. What is important for the sustained implementation of evidence-based brief psychotherapy interventions in psychiatric care? A quantitative evaluation of a real-world programme. Nordic Journal of Psychiatry. 2019;73(3):185-94.

204. Lindow JC, Hughes JL, South C, Gutierrez L, Bannister E, Trivedi MH, et al. Feasibility and Acceptability of the Youth Aware of Mental Health (YAM) Intervention in US Adolescents. Archives of Suicide Research. 2020;24(2):269-84.

205. Locke J, Kang‐Yi C, Pellecchia M, Mandell DS. It's messy but real: a pilot study of the implementation of a social engagement intervention for children with autism in schools. J Res Spec Educ Needs. 2019;19(2):135-44.

206. Locke J, Shih W, Kang-Yi CD, Caramanico J, Shingledecker T, Gibson J, et al. The impact of implementation support on the use of a social engagement intervention for children with autism in public schools. Autism. 2019;23(4):834-45.

207. Loeckx M, Rabinovich RA, Demeyer H, Louvaris Z, Tanner R, Rubio N, et al. Smartphone-Based Physical Activity Telecoaching in Chronic Obstructive Pulmonary Disease: Mixed-Methods Study on Patient Experiences and Lessons for Implementation. JMIR Mhealth Uhealth. 2018;6(12):e200.

208. Long JC, Winata T, Debono D, Phan-Thien K-C, Zhu C, Taylor N. Process evaluation of a behaviour change approach to improving clinical practice for detecting hereditary cancer. BMC Health Serv Res. 2019;19(1):180.

209. Lorthios-Guilledroit A, Filiatrault J, Richard L. What are the factors associated with the implementation of a peer-led health promotion program? Insights from a multiple-case study. Health Education Research. 2019;34(6):578-91.

210. Loucks EB, Nardi WR, Gutman R, Kronish IM, Saadeh FB, Li Y, et al. Mindfulness-Based Blood Pressure Reduction (MB-BP): Stage 1 single-arm clinical trial. PLoS ONE. 2019;14(11):e0223095.

211. Lounsbury DW, Mitchell SG, Dusek KA, Li JZ, Kirk AS, Oros M, et al. Application of System Dynamics to Inform a Model of Adolescent SBIRT Implementation in Primary Care Settings. J Behav Health Serv Res. 2020;47(2):230-44.

212. Lovell M, Birch M-R, Luckett T, Phillips J, Davidson PM, Agar M, et al. Screening and Audit as Service-Level Strategies to Support Implementation of Australian Guidelines for Cancer Pain Management in Adults: A Feasibility Study. Pain Management Nursing. 2019;20(2):113-7.

213. Lum HD, Brungardt A, Jordan SR, Phimphasone-Brady P, Schilling LM, Lin C-T, et al. Design and Implementation of Patient Portal–Based Advance Care Planning Tools. Journal of Pain and Symptom Management. 2019;57(1):112-7.e2.

214. Lyon AR, Bruns EJ, Ludwig K, Vander Stoep A, Pullmann MD, Dorsey S, et al. The Brief Intervention for School Clinicians (BRISC): A Mixed-Methods Evaluation of Feasibility, Acceptability, and Contextual Appropriateness. School Mental Health. 2015;7(4):273-86.

215. Lyon AR, Cook CR, Locke J, Davis C, Powell BJ, Waltz TJ. Importance and feasibility of an adapted set of implementation strategies in schools. Journal of School Psychology. 2019;76:66-77.

216. Lyon AR, Ludwig K, Romano E, Koltracht J, Vander Stoep A, McCauley E. Using Modular Psychotherapy in School Mental Health: Provider Perspectives on Intervention-Setting Fit. Journal of Clinical Child & Adolescent Psychology. 2014;43(6):890-901.

217. Macheel C, Reicks P, Sybrant C, Evans C, Farhat J, West MA, et al. Clinical Decision Support Intervention for Rib Fracture Treatment. Journal of the American College of Surgeons. 2020;231(2):249-56e2.

218. MacPherson HA, Leffler JM, Fristad MA. Implementation of multi-family psychoeducational psychotherapy for childhood mood disorders in an outpatient community setting. J Marital Fam Ther. 2014;40(2):193-211.

219. MacPherson HA, Mackinaw-Koons B, Leffler JM, Fristad MA. Pilot effectiveness evaluation of community-based multi-family psychoeducational psychotherapy for childhood mood disorders. Couple and Family Psychology: Research and Practice. 2016;5(1):43-59.

220. Magge H, Kiflie A, Nimako K, Brooks K, Sodzi-Tettey S, Mobisson-Etuk N, et al. The Ethiopia healthcare quality initiative: design and initial lessons learned. International Journal for Quality in Health Care. 2019;31(10):G180-G6.

221. Magge H, Nahimana E, Mugunga JC, Nkikabahizi F, Tadiri E, Sayinzoga F, et al. The All Babies Count Initiative: Impact of a Health System Improvement Approach on Neonatal Care and Outcomes in Rwanda. Global Health: Science and Practice. 2020;8(3):000-.

222. Magidson JF, Andersen LS, Satinsky EN, Myers B, Kagee A, Anvari M, et al. “Too much boredom isn’t a good thing”: Adapting behavioral activation for substance use in a resource-limited South African HIV care setting. Psychotherapy. 2020;57(1):107-18.

223. Mahmood A, Blaizy V, Verma A, Stephen Sequeira J, Saha D, Ramachandran S, et al. Acceptability and Attitude towards a Mobile-Based Home Exercise Program among Stroke Survivors and Caregivers: A Cross-Sectional Study. International Journal of Telemedicine and Applications. 2019;2019:1-6.

224. Makelarski JA, DePumpo M, Boyd K, Brown T, Kho A, Navalkha C, et al. Implementation of Systematic Community Resource Referrals at Small Primary Care Practices to Promote Cardiovascular Disease Self-Management. J Healthc Qual. 2020;42(5):278-86.

225. Malenga T, Kabaghe AN, Manda-Taylor L, Kadama A, McCann RS, Phiri KS, et al. Malaria control in rural Malawi: implementing peer health education for behaviour change. Global Health. 2017;13(1):84.

226. Margolies PJ, Humensky JL, Chiang IC, Covell NH, Broadway-Wilson K, Gregory R, et al. Is There a Role for Fidelity Self-Assessment in the Individual Placement and Support Model of Supported Employment? Psychiatr Serv. 2017;68(9):975-8.

227. Martin SL, Omotayo MO, Chapleau GM, Stoltzfus RJ, Birhanu Z, Ortolano SE, et al. Adherence partners are an acceptable behaviour change strategy to support calcium and iron-folic acid supplementation among pregnant women in Ethiopia and Kenya: Acceptability of adherence partners to support micronutrient supplementation. Maternal & Child Nutrition. 2017;13(3):e12331.

228. Maxwell AE, Danao LL, Cayetano RT, Crespi CM, Bastani R. Adoption of an evidence-based colorectal cancer screening promotion program by community organizations serving Filipino Americans. BMC Public Health. 2014;14(1):246.

229. McCusker J, Lambert SD, Yaffe MJ, Cole MG, Hidalgo M, Amir E, et al. Pilot Study of a Transitional Intervention for Family Caregivers of Older Adults. Can J Aging. 2019;38(02):210-21.

230. McGuire AB, White DA, Bartholomew T, Flanagan ME, McGrew JH, Rollins AL, et al. The Relationship Between Provider Competence, Content Exposure, and Consumer Outcomes in Illness Management and Recovery Programs. Adm Policy Ment Health. 2017;44(1):81-91.

231. McIntyre T-L, Elkonin D, De Kooker M, Magidson JF. The Application of Mindfulness for Individuals Living with HIV in South Africa: a Hybrid Effectiveness-Implementation Pilot Study. Mindfulness. 2018;9(3):871-83.

232. McKinnon B, Sall M, Vandermorris A, Traoré M, Lamesse-Diedhiou F, McLaughlin K, et al. Feasibility and preliminary effectiveness of group antenatal care in Senegalese health posts: a pilot implementation trial. Health Policy and Planning. 2020;35(5):587-99.

233. McLeod BD, Southam-Gerow MA, Jensen-Doss A, Hogue A, Kendall PC, Weisz JR. Benchmarking Treatment Adherence and Therapist Competence in Individual Cognitive-Behavioral Treatment for Youth Anxiety Disorders. J Clin Child Adolesc Psychol. 2019;48(sup1):S234-S46.

234. Messemaker A, Schall A, Haberstroh J, Pantel J. MultiTANDEM: Training the Trainer to Improve Homecare for People with Dementia. GeroPsych. 2017;30(4):165-75.

235. Miguel-Esponda G, Bohm-Levine N, Rodríguez-Cuevas FG, Cohen A, Kakuma R. Implementation process and outcomes of a mental health programme integrated in primary care clinics in rural Mexico: a mixed-methods study. Int J Ment Health Syst. 2020;14(1):21.

236. Milette-Winfree M, Nakamura BJ, Kotte A, Higa-McMillan C. Multilevel Predictors of Case Managers’ Assessment Administration Behavior in a Precursor to a Measurement Feedback System. Adm Policy Ment Health. 2019;46(5):636-48.

237. Miller CJ, Griffith KN, Stolzmann K, Kim B, Connolly SL, Bauer MS. An Economic Analysis of the Implementation of Team-based Collaborative Care in Outpatient General Mental Health Clinics. Medical Care. 2020;58(10):874-80.

238. Mitchell SG, Gryczynski J, Schwartz RP, Kirk AS, Dusek K, Oros M, et al. Adolescent SBIRT implementation: Generalist vs. Specialist models of service delivery in primary care. Journal of Substance Abuse Treatment. 2020;111:67-72.

239. Mitchell SG, Monico LB, Gryczynski J, O’Grady KE, Schwartz RP. Staff Views of Acceptability and Appropriateness of a Computer-Delivered Brief Intervention for Moderate Drug and Alcohol Use. Journal of Psychoactive Drugs. 2015;47(4):301-7.

240. Mohr DC, Rosen CS, Schnurr PP, Orazem RJ, Noorbaloochi S, Clothier BA, et al. The Influence of Team Functioning and Workload on Sustainability of Trauma-Focused Evidence-Based Psychotherapies. PS. 2018;69(8):879-86.

241. Moreno JHR, Romero Vergara AJ, De Moya DDJDA, Jaramillo Rojas HJ, Díaz Rojas CM, Ciapponi A. [Evaluation of tools for the implementation of clinical practice guidelines on sexually transmitted infections]. Rev Panam Salud Publica. 2017;41:e49.

242. Moretto N, Comans TA, Chang AT, O’Leary SP, Osborne S, Carter HE, et al. Implementation of simulation modelling to improve service planning in specialist orthopaedic and neurosurgical outpatient services. Implementation Science. 2019;14(1):78.

243. Morrel-Samuels S, Rupp LA, Eisman AB, Miller AL, Stoddard SA, Franzen SP, et al. Measuring the Implementation of Youth Empowerment Solutions. Health Promotion Practice. 2018;19(4):581-9.

244. Moser T, Edwards J, Pryor F, Manson L, O'Hare C. Workflow Improvement and the Use of PDSA Cycles: An Exploration Using Screening, Brief Intervention, and Referral to Treatment (SBIRT) Integration. Quality Management in Health Care. 2020;29(2):100-8.

245. Mosson R, Augustsson H, Bäck A, Åhström M, von Thiele Schwarz U, Richter A, et al. Building implementation capacity (BIC): a longitudinal mixed methods evaluation of a team intervention. BMC Health Serv Res. 2019;19(1):287.

246. Mueller K, Naganathan S, Griffey R. Counseling on Access to Lethal Means-Emergency Department (CALM-ED): A Quality Improvement Program for Firearm Injury Prevention. WestJEM. 2020;21(5).

247. Mufson L, Rynn M, Yanes-Lukin P, Choo TH, Soren K, Stewart E, et al. Stepped Care Interpersonal Psychotherapy Treatment for Depressed Adolescents: A Pilot Study in Pediatric Clinics. Adm Policy Ment Health. 2018;45(3):417-31.

248. Mukred M, Yusof ZM, Alotaibi FM. Ensuring the Productivity of Higher Learning Institutions Through Electronic Records Management System (ERMS). IEEE Access. 2019;7:97343-64.

249. Murray H. Evaluation of a Trauma-Focused CBT Training Programme for IAPT services. Behav Cogn Psychother. 2017;45(5):467-82.

250. Myers B, Govender R, Manderscheid R, Williams PP, Johnson K, Koch JR. Need for and Readiness to Implement a Performance Measurement System for South Africa’s Substance Abuse Treatment Services. Int J Ment Health Addiction. 2017;15(4):795-800.

251. Myers B, Sorsdahl K, Morojele NK, Kekwaletswe C, Shuper PA, Parry CDH. “In this thing I have everything I need”: perceived acceptability of a brief alcohol-focused intervention for people living with HIV. AIDS Care. 2017;29(2):209-13.

252. Nadeem E, Saldana L, Chapman J, Schaper H. A Mixed Methods Study of the Stages of Implementation for an Evidence-Based Trauma Intervention in Schools. Behavior Therapy. 2018;49(4):509-24.

253. Naef R, Ernst J, Petry H. Adaption, benefit and quality of care associated with primary nursing in an acute inpatient setting: A cross‐sectional descriptive study. J Adv Nurs. 2019;75(10):2133-43.

254. Neal JW, Neal ZP, Barrett CA, Brutzman B. Are Principals’ Social Networks Associated with Interventions’ Social Validity? School Mental Health. 2020;12(4):812-25.

255. Nelson G, Stefancic A, Rae J, Townley G, Tsemberis S, Macnaughton E, et al. Early implementation evaluation of a multi-site housing first intervention for homeless people with mental illness: A mixed methods approach. Evaluation and Program Planning. 2014;43:16-26.

256. Nguyen AJ, Rykiel N, Murray L, Amin A, Haroz E, Lee C, et al. Stakeholder perspectives on integration of mental health services into primary care: a mixed methods study in Northern Iraq. Int J Ment Health Syst. 2019;13(1):75.

257. Niec LN, Abrahamse ME, Egan R, Coelman FJG, Heiner WD. Global dissemination of parent-child interaction therapy: The perspectives of Dutch trainees. Children and Youth Services Review. 2018;94:485-92.

258. Nunn A, Towey C, Chan PA, Parker S, Nichols E, Oleskey P, et al. Routine HIV Screening in an Urban Community Health Center: Results from a Geographically Focused Implementation Science Program. Public Health Rep. 2016;131(1_suppl):30-40.

259. Nylén EC, Lindfors P, Ishäll L, Göransson S, Aronsson G, Kylin C, et al. A pilot-study of a worksite based participatory intervention program: Its acceptability and short-term effects on work climate and attitudes in human service employees. WOR. 2017;56(4):625-36.

260. O’Dea B, King C, Subotic-Kerry M, Achilles MR, Cockayne N, Christensen H. Smooth Sailing: A Pilot Study of an Online, School-Based, Mental Health Service for Depression and Anxiety. Front Psychiatry. 2019;10:574.

261. Oats RG, Cross WF, Alex Mason W, Casey-Goldstein M, Thompson RW, Hanson K, et al. Implementation assessment of widely used but understudied prevention programs: An illustration from the Common Sense Parenting trial. Evaluation and Program Planning. 2014;44:89-97.

262. Ober AJ, Watkins KE, Hunter SB, Ewing B, Lamp K, Lind M, et al. Assessing and improving organizational readiness to implement substance use disorder treatment in primary care: findings from the SUMMIT study. BMC Fam Pract. 2017;18(1):107.

263. Oftedal S, Burrows T, Fenton S, Murawski B, Rayward AB, Duncan MJ. Feasibility and Preliminary Efficacy of an m-Health Intervention Targeting Physical Activity, Diet, and Sleep Quality in Shift-Workers. IJERPH. 2019;16(20):3810.

264. Okamura KH, Benjamin Wolk CL, Kang-Yi CD, Stewart R, Rubin RM, Weaver S, et al. The Price per Prospective Consumer of Providing Therapist Training and Consultation in Seven Evidence-Based Treatments within a Large Public Behavioral Health System: An Example Cost-Analysis Metric. Front Public Health. 2018;5:356.

265. Onono M, Abdi M, Opondo I, Okung'u J, Asadhi E, Nyamai R, et al. Using the RE-AIM framework to evaluate the implementation of integrated community case management in Kenya. Acta Paediatr. 2018;107:53-62.

266. Pack AP, Golin CE, Hill LM, Carda-Auten J, Wallace DD, Cherkur S, et al. Patient and clinician perspectives on optimizing graphical displays of longitudinal medication adherence data. Patient Education and Counseling. 2019;102(6):1090-7.

267. Palinkas LA, Um MY, Aarons GA, Rafful C, Chavarin CV, Mendoza DV, et al. Implementing Evidence-Based HIV Prevention for Female Sex Workers in Mexico: Provider Assessments of Feasibility and Acceptability. Glob Soc Welf. 2019;6(2):57-68.

268. Paloma V, Morena I, López‐Torres C. Promoting posttraumatic growth among the refugee population in Spain: A community‐based pilot intervention. Health Soc Care Community. 2020;28(1):127-36.

269. Parhiala P, Ranta K, Gergov V, Kontunen J, Law R, La Greca AM, et al. Interpersonal Counseling in the Treatment of Adolescent Depression: A Randomized Controlled Effectiveness and Feasibility Study in School Health and Welfare Services. School Mental Health. 2020;12(2):265-83.

270. Parker BL, Achilles MR, Subotic-Kerry M, O’Dea B. Youth StepCare: a pilot study of an online screening and recommendations service for depression and anxiety among youth patients in general practice. BMC Fam Pract. 2020;21(1):2.

271. Parsons D, Wilson NJ, Vaz S, Lee H, Cordier R. Appropriateness of the TOBY Application, an iPad Intervention for Children with Autism Spectrum Disorder: A Thematic Approach. J Autism Dev Disord. 2019;49(10):4053-66.

272. Parvez SM, Azad R, Rahman M, Unicomb L, Ram PK, Naser AM, et al. Achieving optimal technology and behavioral uptake of single and combined interventions of water, sanitation hygiene and nutrition, in an efficacy trial (WASH benefits) in rural Bangladesh. Trials. 2018;19(1):358.

273. Pérez D, Van Der Stuyft P, Toledo ME, Ceballos E, Fabré F, Lefèvre P. Insecticide treated curtains and residual insecticide treatment to control Aedes aegypti: An acceptability study in Santiago de Cuba. PLoS Negl Trop Dis. 2018;12(1):e0006115.

274. Perfetti AR, Peifer H, Massa S, Taranti LJD, Choudhary M, Collard M, et al. Mixing Beyond Measure: Integrating Methods in a Hybrid Effectiveness–Implementation Study of Operating Room to Intensive Care Unit Handoffs. Journal of Mixed Methods Research. 2019.

275. Perraudin C, Locca J-F, Rossier C, Bugnon O, Schneider M-P. Implementation of an interprofessional medication adherence program for chronic patients in community pharmacies: how much does it cost for the provider? BMC Health Serv Res. 2019;19(1):15.

276. Peterson AE, Bond GR, Drake RE, McHugo GJ, Jones AM, Williams JR. Predicting the Long-Term Sustainability of Evidence-Based Practices in Mental Health Care: An 8-Year Longitudinal Analysis. J Behav Health Serv Res. 2014;41(3):337-46.

277. Peterson R, Darnell D, Berliner L, Dorsey S, Murray L, Monroe-DeVita M. Implementing Transdiagnostic Cognitive Behavioral Psychotherapy in Adult Public Behavioral Health: A Pilot Evaluation of the Feasibility of the Common Elements Treatment Approach (CETA). J Behav Health Serv Res. 2019;46(2):249-66.

278. Pieper MJC, Achterberg WP, Van Der Steen JT, Francke AL. Implementation of a Stepwise, Multidisciplinary Intervention for Pain and Challenging Behaviour in Dementia (STA OP!): A Process Evaluation. International Journal of Integrated Care. 2018;18(3):15.

279. Pineda R, Heiny E, Roussin J, Nellis P, Bogan K, Smith J. Implementation of the Baby Bridge Program Reduces Timing Between NICU Discharge and Therapy Activation. Journal of Early Intervention. 2020;42(3):275-96.

280. Pons-Vigués M, Berenguera A, Coma-Auli N, March S, Pombo H, Masluk B, et al. Qualitative evaluation of a complex intervention to implement health promotion activities according to healthcare attendees and health professionals: EIRA study (phase II). BMJ Open. 2019;9(3):e023872.

281. Pontoski Taylor K, Cunningham A, Schultz L, Jager‐Hyman S, Sposato R, Evans A, et al. Using a cognitive behavioral framework to train staff serving individuals who experience chronic homelessness. Journal of Community Psychology. 2016;44(5):674-80.

282. Porter AL, Margolis AR, Schoen RR, Staresinic CE, Ray CA, Fletcher CD. Use of an extended INR follow-up interval for Veteran patients in an anticoagulation clinic. J Thromb Thrombolysis. 2017;43(3):318-25.

283. Porter KJ, Brock DJ, Estabrooks PA, Perzynski KM, Hecht ER, Ray P, et al. SIPsmartER delivered through rural, local health districts: adoption and implementation outcomes. BMC Public Health. 2019;19(1):1273.

284. Punches BE, Soliman S, Freiermuth CE, Lane BH, Lyons MS. Emergency Nurse Perceptions of Naloxone Distribution in the Emergency Department. Journal of Emergency Nursing. 2020;46(5):675-81.e1.

285. Purbeck CA, Briggs EC, Tunno AM, Richardson LM, Pynoos RS, Fairbank JA. Trauma-informed measurement-based care for children: Implementation in diverse treatment settings. Psychological Services. 2020;17(3):311-22.

286. Pyatak E, King M, Vigen CLP, Salazar E, Diaz J, Schepens Niemiec SL, et al. Addressing Diabetes in Primary Care: Hybrid Effectiveness-Implementation Study of Lifestyle Redesign® Occupational Therapy. Am J Occup Ther. 2019;73(5):7305185020p1-p12.

287. Rahman M, Ashraf S, Unicomb L, Mainuddin AKM, Parvez SM, Begum F, et al. WASH Benefits Bangladesh trial: system for monitoring coverage and quality in an efficacy trial. Trials. 2018;19(1):360.

288. Ramírez Stege AM, Yarris KE. Culture in <i>la clínica</i> : Evaluating the utility of the Cultural Formulation Interview (CFI) in a Mexican outpatient setting. Transcult Psychiatry. 2017;54(4):466-87.

289. Rankin NM, Collett GK, Brown CM, Shaw TJ, White KM, Beale PJ, et al. Implementation of a lung cancer multidisciplinary team standardised template for reporting to general practitioners: a mixed-method study. BMJ Open. 2017;7(12):e018629.

290. Rauktis ME, Bishop-Fitzpatrick L, Jung N, Pennell J. Family group decision making: Measuring fidelity to practice principles in public child welfare. Children and Youth Services Review. 2013;35(2):287-95.

291. Ravid NL, Zamora K, Rehm R, Okumura M, Takayama J, Kaiser S. Implementation of a multidisciplinary discharge videoconference for children with medical complexity: a pilot study. Pilot Feasibility Stud. 2020;6:27.

292. Reaven J, Blakeley-Smith A, Beattie TL, Sullivan A, Moody EJ, Stern JA, et al. Improving transportability of a cognitive-behavioral treatment intervention for anxiety in youth with autism spectrum disorders: Results from a US–Canada collaboration. Autism. 2015;19(2):211-22.

293. Reynolds SS, McLennon SM, Ebright PR, Murray LL, Bakas T. Program evaluation of neuroscience competency programs to implement evidence-based practices: Program evaluation of EBP programs. J Eval Clin Pract. 2017;23(1):149-55.

294. Rijbroek B, Strating MMH, Huijsman R. Implementation of a solution based approach for child protection: A professionals' perspective. Children and Youth Services Review. 2017;82:337-46.

295. Roberts NA, Janda M, Stover AM, Alexander KE, Wyld D, Mudge A, et al. The utility of the implementation science framework “Integrated Promoting Action on Research Implementation in Health Services” (i-PARIHS) and the facilitator role for introducing patient-reported outcome measures (PROMs) in a medical oncology outpatient department. Qual Life Res. 2021;30(11):3063-71.

296. Rodriguez CI, Levinson A, Patel SR, Rottier K, Zwerling J, Essock S, et al. Acceptability of treatments and services for individuals with hoarding behaviors. Journal of Obsessive-Compulsive and Related Disorders. 2016;11:1-8.

297. Rodríguez S, Sanz AM, Llano G, Navarro A, Parra-Lara LG, Krystosik AR, et al. Acceptability and usability of a mobile application for management and surveillance of vector-borne diseases in Colombia: An implementation study. PLoS ONE. 2020;15(5):e0233269.

298. Rohweder C, Wangen M, Black M, Dolinger H, Wolf M, O'Reilly C, et al. Understanding quality improvement collaboratives through an implementation science lens. Preventive Medicine. 2019;129:105859.

299. Romney S, Israel N, Zlatevski D. Exploration-Stage Implementation Variation: Its Effect on the Cost-Effectiveness of an Evidence-Based Parenting Program. Zeitschrift für Psychologie. 2014;222(1):37-48.

300. Rosen NO, Muise MD, Vannier SA, Chambers CT, Scott H, the #postbabyhankypanky Advisory T, et al. #postbabyhankypanky: An Empirically Based Knowledge Sharing Initiative About Sex and the Transition to Parenthood. Arch Sex Behav. 2021;50(1):45-55.

301. Roundfield KD, Lang JM. Costs to Community Mental Health Agencies to Sustain an Evidence-Based Practice. PS. 2017;68(9):876-82.

302. Rowe C, Rigter H, Henderson C, Gantner A, Mos K, Nielsen P, et al. Implementation fidelity of Multidimensional Family Therapy in an international trial. Journal of Substance Abuse Treatment. 2013;44(4):391-9.

303. Roy M, Bolton-Moore C, Sikazwe I, Mukumbwa-Mwenechanya M, Efronson E, Mwamba C, et al. Participation in adherence clubs and on-time drug pickup among HIV-infected adults in Zambia: A matched-pair cluster randomized trial. PLoS Med. 2020;17(7):e1003116.

304. Rye M, Rognmo K, Aarons GA, Skre I. Attitudes Towards the Use of Routine Outcome Monitoring of Psychological Therapies Among Mental Health Providers: The EBPAS–ROM. Adm Policy Ment Health. 2019;46(6):833-46.

305. Sabben G, Mudhune V, Ondeng'e K, Odero I, Ndivo R, Akelo V, et al. A Smartphone Game to Prevent HIV Among Young Africans (Tumaini): Assessing Intervention and Study Acceptability Among Adolescents and Their Parents in a Randomized Controlled Trial. JMIR Mhealth Uhealth. 2019;7(5):e13049.

306. Salahuddin M, Barlow SE, Pont SJ, Butte NF, Hoelscher DM. Development and use of an index for measuring implementation of a weight management program in children in primary care clinics in Texas. BMC Fam Pract. 2018;19(1):191.

307. Saldana L, Chamberlain P, Bradford WD, Campbell M, Landsverk J. The cost of implementing new strategies (COINS): A method for mapping implementation resources using the stages of implementation completion. Children and Youth Services Review. 2014;39:177-82.

308. Salloum RG, Theis RP, Pbert L, Gurka MJ, Porter M, Lee D, et al. Stakeholder Engagement in Developing an Electronic Clinical Support Tool for Tobacco Prevention in Adolescent Primary Care. Children. 2018;5(12):170.

309. Salvador JG, Bhatt SR, Jacobsohn VC, Maley LA, Alkhafaji RS, Rishel Brakey H, et al. Feasibility and acceptability of an online ECHO intervention to expand access to medications for treatment of opioid use disorder, psychosocial treatments and supports. Substance Abuse. 2021;42(4):610-7.

310. Sami S, Kerber K, Tomczyk B, Amsalu R, Jackson D, Scudder E, et al. “You have to take action”: changing knowledge and attitudes towards newborn care practices during crisis in South Sudan. Reproductive Health Matters. 2017;25(51):124-39.

311. Sankey C, Girard S, Cappe E. Evaluation of the social validity and implementation process of a psychoeducational program for parents of a child with Autism Spectrum Disorder. International Journal of Developmental Disabilities. 2021;67(2):101-11.

312. Santesteban-Echarri O, Hernández-Arroyo L, Rice SM, Güerre-Lobera MJ, Serrano-Villar M, Espín-Jaime JC, et al. Adapting the Brief Coping Cat for children with anxiety to a group setting in the Spanish public mental health system: A hybrid effectiveness-implementation pilot study. Journal of Child and Family Studies. 2018;27:3300-15.

313. Saunders J, Hipple NK, Allison K, Peterson J. Estimating the Impact of Research Practitioner Partnerships on evidence-based Program Implementation*. Justice Quarterly. 2020:1-21.

314. Scheirer MA, Santos SLZ, Tagai EK, Bowie J, Slade J, Carter R, et al. Dimensions of sustainability for a health communication intervention in African American churches: a multi-methods study. Implementation Science. 2017;12(1):43.

315. Schuetz N, Mendenhall AN, Grube W. Strengths Model for Youth Case Management: Impact on the Provider and Agency. Child Adolesc Soc Work J. 2021;38(1):43-55.

316. Seth B, Herbst N, Oleinik K, Clark K, Helm ED, O’Donnell C, et al. Feasibility, Acceptability, and Adoption of an Inpatient Tobacco Treatment Service at a Safety-Net Hospital: A Mixed-Methods Study. Annals ATS. 2020;17(1):63-71.

317. Shaffer KM, Nelson CJ, DuHamel KN. Barriers to participation in a sexual health intervention for men following treatment for rectal and anal cancer. Psycho-Oncology. 2018;27(3):1082-5.

318. Shapiro CJ, Charest E. Factors associated with provider self‐efficacy in delivery of evidence‐based programs for children, youth, and families. Child & Family Social Work. 2020;25(3):637-47.

319. Shea CM, Teal R, Haynes-Maslow L, McIntyre M, Weiner BJ, Wheeler SB, et al. Assessing the feasibility of a virtual tumor board program: a case study. J Healthc Manag. 2014;59(3):177-93.

320. Shire SY, Goods K, Shih W, Distefano C, Kaiser A, Wright C, et al. Parents’ Adoption of Social Communication Intervention Strategies: Families Including Children with Autism Spectrum Disorder Who are Minimally Verbal. J Autism Dev Disord. 2015;45(6):1712-24.

321. Shire SY, Shih W, Chang Y-C, Kasari C. Short Play and Communication Evaluation: Teachers’ assessment of core social communication and play skills with young children with autism. Autism. 2018;22(3):299-310.

322. Sigmarsdóttir M, Forgatch MS, Guðmundsdóttir EV, Thorlacius Ö, Svendsen GT, Tjaden J, et al. Implementing an Evidence-Based Intervention for Children in Europe: Evaluating the Full-Transfer Approach. Journal of Clinical Child & Adolescent Psychology. 2019;48(sup1):S312-S25.

323. Sijercic I, Lane JEM, Gutner CA, Monson CM, Stirman SW. The Association Between Clinician and Perceived Organizational Factors with Early Fidelity to Cognitive Processing Therapy for Posttraumatic Stress Disorder in a Randomized Controlled Implementation Trial. Adm Policy Ment Health. 2020;47(1):8-18.

324. Sijpkens MK, Lagendijk J, Van Minde MRC, De Kroon MLA, Bertens LCM, Rosman AN, et al. Integrating interconception care in preventive child health care services: The Healthy Pregnancy 4 All program. PLoS ONE. 2019;14(11):e0224427.

325. Sinclair KA, Zamora-Kapoor A, Townsend-Ing C, McElfish PA, Kaholokula JK. Implementation outcomes of a culturally adapted diabetes self-management education intervention for Native Hawaiians and Pacific islanders. BMC Public Health. 2020;20(1):1579.

326. Small JW, Frey A, Lee J, Seeley JR, Scott TM, Sibley MH. Fidelity of Motivational Interviewing in School-Based Intervention and Research. Prev Sci. 2021;22(6):712-21.

327. Smith AM, Stewart K, Baul T, Valentine SE. Peer delivery of a brief cognitive‐behavioral treatment for posttraumatic stress disorder: A hybrid effectiveness‐implementation pilot study. Journal of Clinical Psychology. 2020;76(12):2133-54.

328. Soi C, Babigumira JB, Chilundo B, Muchanga V, Matsinhe L, Gimbel S, et al. Implementation strategy and cost of Mozambique’s HPV vaccine demonstration project. BMC Public Health. 2019;19(1):1406.

329. Spector AY, Pinto RM, Rahman R, Da Fonseca A. Implementation of Brazil's “family health strategy”: Factors associated with community health workers’, nurses’, and physicians’ delivery of drug use services. International Journal of Drug Policy. 2015;26(5):509-15.

330. Stahmer AC, Rieth S, Lee E, Reisinger EM, Mandell DS, Connell JE. TRAINING TEACHERS TO USE EVIDENCE-BASED PRACTICES FOR AUTISM: EXAMINING PROCEDURAL IMPLEMENTATION FIDELITY: Training Teachers in Autism Practices. Psychology in the Schools. 2015;52(2):181-95.

331. Stamatakis KA, McQueen A, Filler C, Boland E, Dreisinger M, Brownson RC, et al. Measurement properties of a novel survey to assess stages of organizational readiness for evidence-based interventions in community chronic disease prevention settings. Implementation Science. 2012;7(1):65.

332. Stevens BJ, Yamada J, Promislow S, Barwick M, Pinard M, Pain CTiCs. Pain Assessment and Management After a Knowledge Translation Booster Intervention. Pediatrics. 2016;138(4):e20153468.

333. Stockdale SE, Hamilton AB, Bergman AA, Rose DE, Giannitrapani KF, Dresselhaus TR, et al. Assessing fidelity to evidence-based quality improvement as an implementation strategy for patient-centered medical home transformation in the Veterans Health Administration. Implementation Science. 2020;15(1):18.

334. Suhrheinrich J, Rieth SR, Dickson KS, Stahmer AC. Exploring Associations Between Inner-Context Factors and Implementation Outcomes. Exceptional Children. 2020;86(2):155-73.

335. Sutherland KS, Conroy MA, McLeod BD, Algina J, Kunemund RL. Factors Associated with Teacher Delivery of a Classroom-Based Tier 2 Prevention Program. Prev Sci. 2018;19(2):186-96.

336. Sutherland KS, Conroy MA, McLeod BD, Algina J, Wu E. Teacher Competence of Delivery of BEST in CLASS as a Mediator of Treatment Effects. School Mental Health. 2018;10(3):214-25.

337. Swain DM, Winter J, Klein CB, Lemelman A, Giordano J, Jablon NN, et al. Augmented naturalistic developmental behavioral intervention for toddlers with autism spectrum disorder: A community pilot study. International Review of Research in Developmental Disabilities. 2020;59:47-70.

338. Swindle T, Selig JP, Rutledge JM, Whiteside-Mansell L, Curran G. Fidelity monitoring in complex interventions: a case study of the WISE intervention. Arch Public Health. 2018;76(1):53.

339. Szeszulski J, Walker T, Robertson M, Cuccaro P, Fernandez ME. School Staff's Perspectives on the Adoption of Elementary-School Physical Activity Approaches: A Qualitative Study. Am J Health Educ. 2020;51(6):395-405.

340. Tabak RG, Dsouza N, Schwarz CD, Quinn K, Kristen P, Haire-Joshu D. A formative study to understand perspectives of families eligible for a pediatric obesity program: a qualitative study. BMC Public Health. 2018;18(1):586.

341. Taylor LJ, Adkins S, Hoel AW, Hauser J, Suwanabol P, Wood G, et al. Using Implementation Science to Adapt a Training Program to Assist Surgeons with High-Stakes Communication. Journal of Surgical Education. 2019;76(1):165-73.

342. Théodore FL, Bonvecchio Arenas A, García-Guerra A, García IB, Alvarado R, Rawlinson CJ, et al. Sociocultural Influences on Poor Nutrition and Program Utilization of Mexico's Conditional Cash Transfer Program. The Journal of Nutrition. 2019;149:2290S-301S.

343. Thies K, Schiessl A, Khalid N, Hess AM, Harding K, Ward D. Evaluation of a learning collaborative to advance team-based care in Federally Qualified Health Centers. BMJ Open Qual. 2020;9(3):e000794.

344. Thompson T, Kreuter MW, Caito N, Williams RS, Escoffery C, Fernandez ME, et al. Implementing an Evidence-based Tobacco Control Program at Five 2-1-1 Call Centers: An Evaluation Using the Consolidated Framework for Implementation Research. Nicotine & Tobacco Research. 2019;21(2):180-7.

345. Thompson-Brenner H, Boswell JF, Espel-Huynh H, Brooks G, Lowe MR. Implementation of transdiagnostic treatment for emotional disorders in residential eating disorder programs: A preliminary pre-post evaluation. Psychotherapy Research. 2019;29(8):1045-61.

346. Tinc PJ, Jenkins P, Sorensen JA, Weinehall L, Gadomski A, Lindvall K. Key factors for successful implementation of the National Rollover Protection Structure Rebate Program: A correlation analysis using the consolidated framework for implementation research. Scand J Work Environ Health. 2020;46(1):85-95.

347. Toles M, Leeman J, Colón-Emeric C, Hanson LC. Implementing a Standardized Transition Care Plan in Skilled Nursing Facilities. J Appl Gerontol. 2020;39(8):855-62.

348. Tollefsen TK, Darrow SM, Lohne V, Berg-Nielsen TS. Experiences with using an idiographic assessment procedure in primary mental health care services for adolescents. International Journal of Qualitative Studies on Health and Well-being. 2020;15(1):1763741.

349. Topp SM, Chetty-Makkan CM, Smith HJ, Chimoyi L, Hoffmann CJ, Fielding K, et al. “It's Not Like Taking Chocolates”: Factors Influencing the Feasibility and Sustainability of Universal Test and Treat in Correctional Health Systems in Zambia and South Africa. Global Health: Science and Practice. 2019;7(2):189-202.

350. Toyserkani GA, Huynh L, Morrato EH. Adaptation for Regulatory Application: A Content Analysis of FDA Risk Evaluation and Mitigation Strategies Assessment Plans (2014–2018) Using RE-AIM. Front Public Health. 2020;8:43.

351. Tutelman PR, Chambers CT, Stinson JN, Parker JA, Barwick M, Witteman HO, et al. The Implementation Effectiveness of a Freely Available Pediatric Cancer Pain Assessment App: A Pilot Implementation Study. JMIR Cancer. 2018;4(2):e10280.

352. Vaccaro L, Shaw J, Sethi S, Kirsten L, Beatty L, Mitchell G, et al. Barriers and facilitators to community‐based psycho‐oncology services: A qualitative study of health professionals’ attitudes to the feasibility and acceptability of a shared care model. Psycho-Oncology. 2019;28(9):1862-70.

353. Valentine SE, Ahles EM, Dixon De Silva LE, Patrick KA, Baldwin M, Chablani-Medley A, et al. Community-Based Implementation of a Paraprofessional-Delivered Cognitive Behavioral Therapy Program for Youth Involved with the Criminal Justice System. Journal of Health Care for the Poor and Underserved. 2019;30(2):841-65.

354. Valentine SE, Borba CPC, Dixon L, Vaewsorn AS, Guajardo JG, Resick PA, et al. Cognitive Processing Therapy for Spanish-speaking Latinos: A Formative Study of a Model-Driven Cultural Adaptation of the Manual to Enhance Implementation in a Usual Care Setting: Adaptation of CPT for Spanish-Speaking Latinos. Journal of Clinical Psychology. 2017;73(3):239-56.

355. Van De Mortel TF, Armit L, Shanahan B, Needham J, Brown C, Grafton E, et al. Supporting Australian clinical learners in a collaborative clusters education model: a mixed methods study. BMC Nurs. 2020;19(1):57.

356. Van Der Giessen JAM, Ausems MGEM, Van Den Muijsenbergh METC, Van Dulmen S, Fransen MP. Systematic development of a training program for healthcare professionals to improve communication about breast cancer genetic counseling with low health literate patients. Familial Cancer. 2020;19(4):281-90.

357. Van Der Kleij RMJJ, Crone MR, Reis R, Paulussen TGWM. Critical stakeholder determinants to the implementation of intersectoral community approaches targeting childhood obesity. Health Education Research. 2016;31(6):697-715.

358. Van Der Westhuizen C, Myers B, Malan M, Naledi T, Roelofse M, Stein DJ, et al. Implementation of a screening, brief intervention and referral to treatment programme for risky substance use in South African emergency centres: A mixed methods evaluation study. PLoS ONE. 2019;14(11):e0224951.

359. Vides-Porras A, Cáceres P, Company A, Guillen O, Arrien MA, Castellano Y, et al. Gaining insight into the implementation of an e-learning smoking cessation course in Latin American countries. Health Promotion International. 2021;36(2):349-62.

360. Viljoen JL, Cochrane DM, Shaffer CS, Muir NM, Brodersen EM, Rogers BJ, et al. Bridging Risk Assessments to Case Planning: Development and Evaluation of an Intervention-Planning Tool for Adolescents on Probation. Criminal Justice and Behavior. 2019;46(11):1587-610.

361. Visher CA, Hiller M, Belenko S, Pankow J, Dembo R, Frisman LK, et al. The Effect of a Local Change Team Intervention on Staff Attitudes Towards HIV Service Delivery in Correctional Settings: A Randomized Trial. AIDS Education and Prevention. 2014;26(5):411-28.

362. Vogel RGM, Bours GJJW, Metzelthin SF, Erkens PMG, Van Breukelen GJP, Zwakhalen SMG, et al. The perceived behavior and barriers of community care professionals in encouraging functional activities of older adults: the development and validation of the MAINtAIN-C questionnaire. BMC Health Serv Res. 2020;20(1):907.

363. Vousden N, Lawley E, Seed PT, Gidiri MF, Charantimath U, Makonyola G, et al. Exploring the effect of implementation and context on a stepped-wedge randomised controlled trial of a vital sign triage device in routine maternity care in low-resource settings. Implement Sci. 2019;14:38.

364. Wainer AL, Pickard K, Ingersoll BR. Using Web-Based Instruction, Brief Workshops, and Remote Consultation to Teach Community-Based Providers a Parent-Mediated Intervention. Journal of Child and Family Studies. 2017;26(6):1592-602.

365. Wambiya EOA, Atela M, Eboreime E, Ibisomi L. Factors affecting the acceptability of isoniazid preventive therapy among healthcare providers in selected HIV clinics in Nairobi County, Kenya: a qualitative study. BMJ Open. 2018;8(12):e024286.

366. Wang B, Stanton B, Lunn S, Patel P, Koci V, Deveaux L. Development of a Brief Pre-Implementation Screening Tool to Identify Teachers Who Are at Risk for Not Implementing Intervention Curriculum and High-Implementing Teachers. Health Educ Behav. 2017;44(1):83-91.

367. Ware P, Ross HJ, Cafazzo JA, Laporte A, Gordon K, Seto E. Evaluating the Implementation of a Mobile Phone–Based Telemonitoring Program: Longitudinal Study Guided by the Consolidated Framework for Implementation Research. JMIR Mhealth Uhealth. 2018;6(7):e10768.

368. Weiner BJ, Lewis CC, Stanick C, Powell BJ, Dorsey CN, Clary AS, et al. Psychometric assessment of three newly developed implementation outcome measures. Implementation Science. 2017;12(1):108.

369. Weiner D, Navalkha C, Abramsohn E, DePumpo M, Paradise K, Stiehl M, et al. Mobile resource referral technology for preventive child welfare services: Implementation and feasibility. Children and Youth Services Review. 2019;107:104499.

370. Welsh WN, Lin H-J, Peters RH, Stahler GJ, Lehman WEK, Stein LAR, et al. Effects of a strategy to improve offender assessment practices: Staff perceptions of implementation outcomes. Drug and Alcohol Dependence. 2015;152:230-8.

371. Whitaker K, Fortier A, Bruns EJ, Nicodimos S, Ludwig K, Lyon AR, et al. How Do School Mental Health Services Vary Across Contexts? Lessons Learned from Two Efforts to Implement a Research-Based Strategy. School Mental Health. 2018;10(2):134-46.

372. White MC, Daya L, Karel FKB, White G, Abid S, Fitzgerald A, et al. Using the Knowledge to Action Framework to Describe a Nationwide Implementation of the WHO Surgical Safety Checklist in Cameroon. Anesthesia & Analgesia. 2020;130(5):1425-34.

373. White MC, Randall K, Capo-Chichi NFE, Sodogas F, Quenum S, Wright K, et al. Implementation and evaluation of nationwide scale-up of the Surgical Safety Checklist. British Journal of Surgery. 2019;106(2):e91-e102.

374. Whiteside LK, Darnell D, Jackson K, Wang J, Russo J, Donovan DM, et al. Collaborative care from the emergency department for injured patients with prescription drug misuse: An open feasibility study. Journal of Substance Abuse Treatment. 2017;82:12-21.

375. Wibowo E, Wassersug RJ, Robinson JW, Santos-Iglesias P, Matthew A, McLeod DL, et al. An Educational Program to Help Patients Manage Androgen Deprivation Therapy Side Effects: Feasibility, Acceptability, and Preliminary Outcomes. Am J Mens Health. 2020;14(1):155798831989899.

376. Wied TS, Poth A, Pantel J, Oswald F, Haberstroh J, Consortium E. How do dementia researchers view support tools for informed consent procedures of persons with dementia? Z Gerontol Geriat. 2021;54(7):667-75.

377. Wieman DA, Camacho-Gonsalves T, Huckshorn KA, Leff S. Multisite Study of an Evidence-Based Practice to Reduce Seclusion and Restraint in Psychiatric Inpatient Facilities. PS. 2014;65(3):345-51.

378. Wijma EM, Veerbeek MA, Prins M, Pot AM, Willemse BM. A virtual reality intervention to improve the understanding and empathy for people with dementia in informal caregivers: results of a pilot study. Aging & Mental Health. 2018;22(9):1121-9.

379. Wilhelm DJ, Brenner S, Muula AS, De Allegri M. A qualitative study assessing the acceptability and adoption of implementing a results based financing intervention to improve maternal and neonatal health in Malawi. BMC Health Serv Res. 2016;16(1):398.

380. Willging CE, Harkness A, Israel T, Ley D, Hokanson PS, DeMaria C, et al. A Mixed-Method Assessment of a Pilot Peer Advocate Intervention for Rural Gender and Sexual Minorities. Community Ment Health J. 2018;54(4):395-409.

381. Williams NJ. Assessing mental health clinicians’ intentions to adopt evidence-based treatments: reliability and validity testing of the evidence-based treatment intentions scale. Implementation Science. 2015;11(1):60.

382. Williams NJ, Glisson C, Hemmelgarn A, Green P. Mechanisms of Change in the ARC Organizational Strategy: Increasing Mental Health Clinicians’ EBP Adoption Through Improved Organizational Culture and Capacity. Adm Policy Ment Health. 2017;44(2):269-83.

383. Williamson E, Srikesavan C, Thompson J, Tonga E, Eldridge L, Adams J, et al. Translating the Strengthening and Stretching for Rheumatoid Arthritis of the Hand Programme from clinical trial to clinical practice: An effectiveness–implementation study. Hand Therapy. 2020;25(3):87-97.

384. Witte SS, Wu E, El-Bassel N, Hunt T, Gilbert L, Medina KP, et al. Implementation of a couple-based HIV prevention program: a cluster randomized trial comparing manual versus Web-based approaches. Implementation Science. 2014;9(1):116.

385. Wolfenden L, Nathan N, Janssen LM, Wiggers J, Reilly K, Delaney T, et al. Multi-strategic intervention to enhance implementation of healthy canteen policy: a randomised controlled trial. Implementation Science. 2017;12(1):6.

386. Wood SM, White K, Peebles R, Pickel J, Alausa M, Mehringer J, et al. Outcomes of a Rapid Adolescent Telehealth Scale-Up During the COVID-19 Pandemic. Journal of Adolescent Health. 2020;67(2):172-8.

387. Yadav K, Chamberlain JM, Lewis VR, Abts N, Chawla S, Hernandez A, et al. Designing Real-time Decision Support for Trauma Resuscitations. Acad Emerg Med. 2015;22(9):1076-84.

388. Yang JP, Simoni JM, Dorsey S, Lin Z, Sun M, Bao M, et al. Reducing distress and promoting resilience: a preliminary trial of a CBT skills intervention among recently HIV-diagnosed MSM in China. AIDS Care. 2018;30(sup5):S39-S48.

389. Yapa HM, De Neve J-W, Chetty T, Herbst C, Post FA, Jiamsakul A, et al. The impact of continuous quality improvement on coverage of antenatal HIV care tests in rural South Africa: Results of a stepped-wedge cluster-randomised controlled implementation trial. PLoS Med. 2020;17(10):e1003150.

390. Youn SJ, Aguilar-Silvan Y, Baldwin M, Chablani-Medley A, Patrick KA, Shtasel DL, et al. Ensuring the fit of an evidence-based curriculum for high-risk Latina young mothers using implementation science. Journal of Community Psychology. 2021;49(2):737-55.

391. Young N, Achieng F, Desai M, Phillips-Howard P, Hill J, Aol G, et al. Integrated point-of-care testing (POCT) for HIV, syphilis, malaria and anaemia at antenatal facilities in western Kenya: a qualitative study exploring end-users’ perspectives of appropriateness, acceptability and feasibility. BMC Health Serv Res. 2019;19(1):74.

392. Young N, Taegtmeyer M, Aol G, Bigogo GM, Phillips-Howard PA, Hill J, et al. Integrated point-of-care testing (POCT) of HIV, syphilis, malaria and anaemia in antenatal clinics in western Kenya: A longitudinal implementation study. PLoS ONE. 2018;13(7):e0198784.

393. Yuan CT, Nembhard IM, Kane GC. The influence of peer beliefs on nurses' use of new health information technology: A social network analysis. Social Science & Medicine. 2020;255:113002.

394. Zakumumpa H, Bennett S, Ssengooba F. Accounting for variations in ART program sustainability outcomes in health facilities in Uganda: a comparative case study analysis. BMC Health Serv Res. 2016;16(1):584.

395. Zakumumpa H, Kwiringira J, Rujumba J, Ssengooba F. Assessing the level of institutionalization of donor-funded anti-retroviral therapy (ART) programs in health facilities in Uganda: implications for program sustainability. Global Health Action. 2018;11(1):1523302.

396. Zandberg LJ, Wilson GT. Train-the-Trainer: Implementation of Cognitive Behavioural Guided Self-Help for Recurrent Binge Eating in a Naturalistic Setting: Implementation of CBTGSH for Recurrent Binge Eating. Eur Eat Disorders Rev. 2013;21(3):230-7.

397. Zimmerman L, Lounsbury DW, Rosen CS, Kimerling R, Trafton JA, Lindley SE. Participatory System Dynamics Modeling: Increasing Stakeholder Engagement and Precision to Improve Implementation Planning in Systems. Adm Policy Ment Health. 2016;43(6):834-49.

398. Zullig LL, McCant F, Silberberg M, Johnson F, Granger BB, Bosworth HB. Changing CHANGE: adaptations of an evidence-based telehealth cardiovascular disease risk reduction intervention. Transl Behav Med. 2018;8(2):225-32.
